# The potential use of nanozymes as an antibacterial agents in oral infection, periodontitis, and peri-implantitis

**DOI:** 10.1186/s12951-024-02472-x

**Published:** 2024-04-25

**Authors:** Mohammad Hosseini Hooshiar, Ashkan Badkoobeh, Shirin Kolahdouz, Azadeh Tadayonfard, Asieh Mozaffari, Kamyar Nasiri, Sara Salari, Reza Safaralizadeh, Saman Yasamineh

**Affiliations:** 1 Department of Periodontology, School of Dentistry, Tehran University of Medical Sciences, Tehran, Iran; 2 Department of Oral and Maxillofacial Surgery, School of Dentistry, Qom University of Medical Sciences, Qom, Iran; 3 School of Dentistry, Shahid Sadoughi University of Medical Sciences, Yazd, Iran; 4 Postgraduate Department of Prosthodontics, Dental Faculty, Tehran University of Medical Sciences, Tehran, Iran; 5 Department of Periodontics, Faculty of Dentistry, Qazvin University of Medical Sciences, Qazvin, Iran; 6 Department of Dentistry, Islamic Azad University of Medical Sciences, Tehran, Iran; 7 Islamic Azad University of Medical Sciences, Esfahan, Iran; 8 Restarative Dentistry, Department of Dental, Faculty Tabriz Medical University, Tabriz, Iran; 9 Young Researchers and Elite Club, Tabriz Branch, Islamic Azad University, Tabriz, Iran

**Keywords:** Nanozymes, Periodontitis, Oral infection, Peri-implantitis, Antibacterial

## Abstract

Several studies suggest that oral pathogenic biofilms cause persistent oral infections. Among these is periodontitis, a prevalent condition brought on by plaque biofilm. It can even result in tooth loss. Furthermore, the accumulation of germs around a dental implant may lead to peri-implantitis, which damages the surrounding bone and gum tissue. Furthermore, bacterial biofilm contamination on the implant causes soft tissue irritation and adjacent bone resorption, severely compromising dental health. On decontaminated implant surfaces, however, re-osseointegration cannot be induced by standard biofilm removal techniques such as mechanical cleaning and antiseptic treatment. A family of nanoparticles known as nanozymes (NZs) comprise highly catalytically active multivalent metal components. The most often employed NZs with antibacterial activity are those that have peroxidase (POD) activity, among other types of NZs. Since NZs are less expensive, more easily produced, and more stable than natural enzymes, they hold great promise for use in various applications, including treating microbial infections. NZs have significantly contributed to studying implant success rates and periodontal health maintenance in periodontics and implantology. An extensive analysis of the research on various NZs and their applications in managing oral health conditions, including dental caries, dental pulp disorders, oral ulcers, peri-implantitis, and bacterial infections of the mouth. To combat bacteria, this review concentrates on NZs that imitate the activity of enzymes in implantology and periodontology. With a view to the future, there are several ways that NZs might be used to treat dental disorders antibacterially.

**Graphical Abstract:**

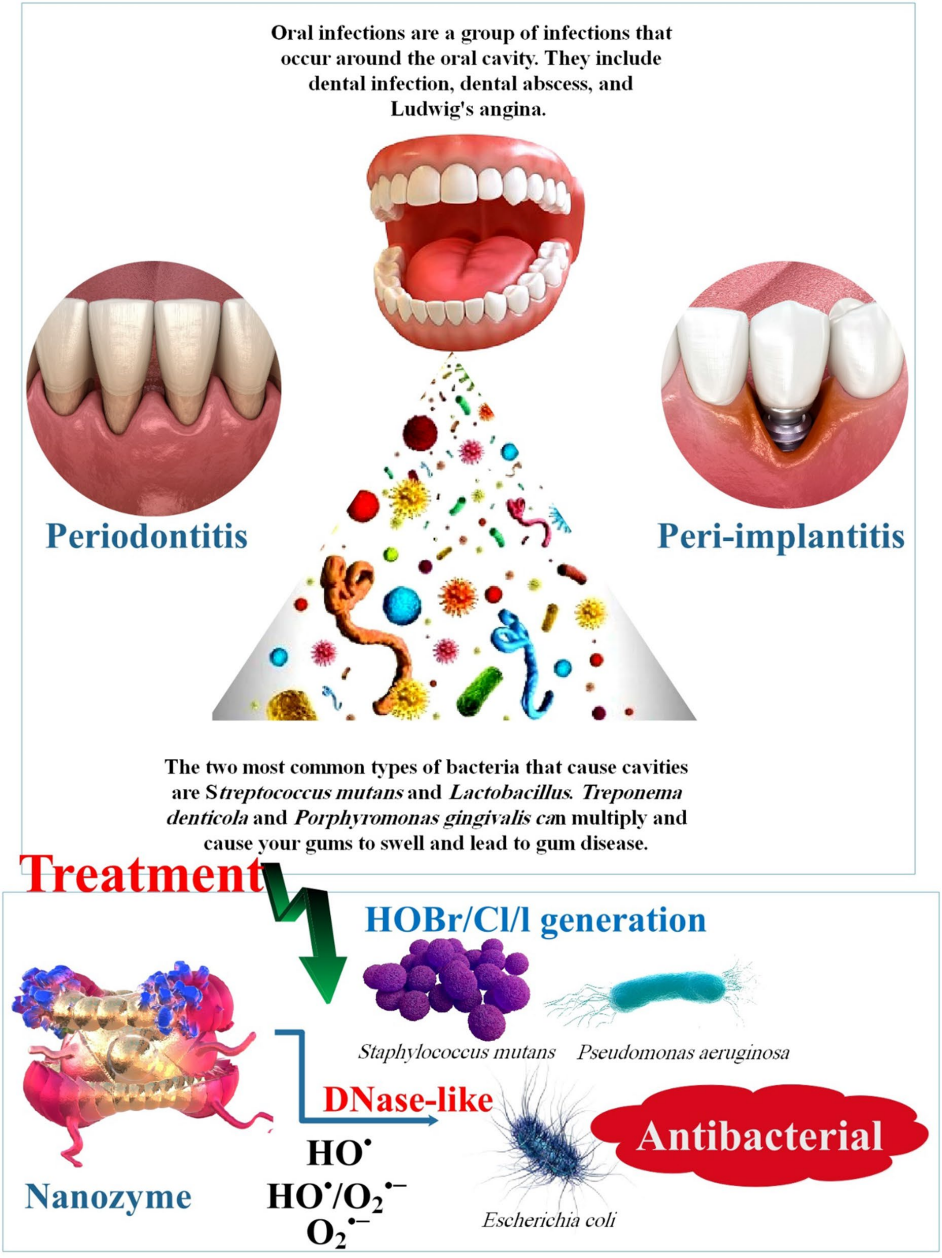

## Introduction

Globally, bacterial infections pose a significant risk to public health. Antibiotics continue to be the most comprehensive form of medical intervention for bacterial infections. Nevertheless, antibiotic abuse and neglect have significantly contributed to the development of antibiotic-resistant strains, most notably in the context of the COVID-19 pandemic. Moreover, antibiotic management strategies are dramatically impacted by the improper and excessive application of disinfectants and biocides. As a result, alternative antibacterial remedies are required immediately to alleviate this crisis. Nanozymes (NZs) have emerged as highly prospective novel antibacterial agents in recent times due to their broad-spectrum antibacterial activity, reduced drug resistance, and exceptional stability [1, 2]. Nanotechnology presents an innovative solution to the most urgent problems of the present day. Nanotechnology applications provide a flawless and accurate alternative in dentistry and appear to have solutions to issues that arise in conventional dental practices. These novel nanoparticles (NPs) can imitate the surface and contact characteristics of tooth tissues. Since the inception and application of NPs and nanocarriers, nanotechnology has been applied extensively in various fields. NPCs may readily breach the defenses of biological organisms due to their diminutive size. The potential applications of nanobiomedical knowledge have also been investigated, encompassing imaging, early-stage disease diagnostics, and the targeted and efficient transportation of pharmaceuticals, DNA, and therapeutic compounds to particular organs or cells [3–6].

Furthermore, there are numerous conventional therapies available to address periodontitis, each of which focuses on a distinct facet of the condition’s etiology and pathogenesis. The use of antibiotics and antimicrobial drugs is prevalent in treatment. Nevertheless, conventional methods are rendered ineffective as a result of drug resistance and the development of adverse effects. Because of their biocompatibility, extended shelf life, and high specific surface area to volume ratio, metal NPs exhibit the most significant potential as antibacterial agents. Many scientists are becoming intrigued by metal NPs due to the development of drug-resistant strains and the enhancement of microbial resistance to antibiotics. The biofilm can be inhibited due to the NPs’ miniature size allowing them to penetrate the biofilm matrix and establish direct contact with the bacterial cells. It is anticipated that antibacterial research will advance further as we approach practical applications. Metal NPs exhibit potent antibacterial properties [7]. Because silver-based biomaterials (AgBMs) have the potential to be very effective antibacterial agents with relatively low toxicity, a great deal of study has been done on them. AgBMs display various antimicrobial properties, including bacterial cell membrane disruption, disruption of bacterial proteins and enzymes, bacterial contact death, and damage to genetic material. More specifically, AgBMs are now more helpful because of improvements in nanotechnology. Consequently, AgBMs have been investigated and used in a wide range of dental subspecialties, such as implant coating, denture additives, periodontal plaque suppression, caries prevention or arrest, root canal sterilization, and anti-inflammatory material in oral and maxillofacial surgery [8].

Since magnetite NPs were shown to have horseradish peroxidase (POD)-like activity in 2007, other researchers have looked into various NP forms that exhibit enzyme-like activities, notably NZs [9]. NZs exhibit superior efficacy, stability, and cost-effectiveness compared to their natural counterparts [10]. As a result, NZs have already been thoroughly investigated in several fields, such as chemical engineering, agriculture, the food industry, dentistry, and medical research [11]. Natural enzymes have been applied extensively in various sectors, such as industry, medicine, biology, and more, owing to their potent catalytic capabilities and substrate specificity. While showing potential, these materials frequently encounter inherent deficiencies, including exorbitant expenses, precarious operational stability, and recycling complexities [12, 13]. For a very long time, researchers have been exploring artificial enzyme mimics as a means of overcoming these inadequacies. One class of nanomaterials having enzyme catalytic characteristics is NZs. Since NZs are less expensive, more stable, and more durable than natural enzymes, they are often used in the biological, medicinal, and industrial domains [14]. A comprehensive understanding of the potential catalytic mechanisms will significantly aid in advancing innovative and highly effective NZs, while the logical control of their activities holds immense importance [15, 16]. Present a comprehensive analysis of the categorization, catalytic process, and regulation of activity, along with recent advancements in research concerning NZs utilized in biosensing, environmental protection, and disease treatment, among other applications, over the last few years [17]. While antibiotic therapy is the most commonly accepted paradigm for treating these kinds of illnesses, long-term overuse, abuse, and misuse of antibiotic-based medications have led to the emergence of super-bacteria that are resistant to several antibiotics [18].

However, due to the numerous limitations of natural enzymes, engineered NZs are increasingly being utilized as viable substitutes in antibacterial therapy that does not involve antibiotics [19]. Because of their high membrane permeability and biocompatibility, NZs are also less prone to acquire bacterial resistance. More significantly, bacterial biofilms may be eliminated by using NZs capable of catalysis [20, 21].

NZs are composed of various materials, including iron-based nanomaterials, carbon dots (CDs), carbon nanotubes (CNTs), graphene oxide, carbon nitride, fullerene, polymer-based substances, noble and non-noble metals, and their derivatives. Their efficacy has been demonstrated across multiple catalytic activity categories, including those of superoxide dismutase (SOD)-like, POD-like, and oxidase (OXD)-like enzymes [22].

An estimated billion people worldwide are impacted by oral disorders, such as dental caries, periodontal disease, and oral cancer, according to a series of articles on oral health published in The Lancet in 2019. As biomaterials evolve quickly, stomatology also advances, significantly advancing the prevention and treatment of oral diseases [23]. Nevertheless, conventional dental materials, including Ag amalgam, possess certain drawbacks that can result in associated complications and ultimately unsuccessful treatments [24, 25]. The development of nanomaterials has opened up a wide range of options for improving oral function, maintaining dental health, and improving overall quality of life. Numerous naturally occurring enzymes, including amylase and proteolytic enzymes, have been proven to have antibacterial, anti-inflammatory, and immunity-boosting properties in oral studies and applications. These enzymes may be utilized to treat dental caries and mouth ulcers [26]. Oral cancer arises from a multitude of factors, including genetic modifications, interactions within the tumor microenvironment (TME), lifestyle choices, and microbial infections that are associated with the disease [27]. Oral cancer is treated and diagnosed using a variety of techniques. Artificial enzymes called NZs have significant promise for the treatment and diagnosis of cancers. Compared to natural enzymes, they are much more advantageous and have unique biological and physical characteristics [19, 28].

Pathogenic biofilm-induced oral diseases, such as periodontitis caused by the accumulation of bacterial biofilm on the gums and teeth, have presented a substantial risk to human health [29]. Conventional therapeutic approaches, including mechanical debridement and antibiotic therapy, demonstrate limited efficacy in treating the condition. In treating oral diseases, numerous NZs with exceptional antibacterial activity have been utilized extensively in recent years [30]. Natural enzymes do, however, have several drawbacks, including poor stability in severe environments (such as heat and extreme pH), high production costs, time-consuming separation and purification, and long-term storage difficulties, among others [31, 32].

The development of dentistry is parallel to that of material science. Oral NZ research and application is emerging as a new subfield of nanocatalytic medicine [33]. To underscore the significant impact of NZs on dental health, an initial examination was conducted of the overall research advancements in multifunctional NZs for the treatment of oral diseases such as dental caries, pulp diseases, ulcers, and peri-implantitis; surveillance of oral cancer, oral bacteria, and ions; and regeneration of both soft and hard tissue [34]. Oral maladies induced by biofilm are treated with a variety of conventional techniques, including mechanical scaling and root planing; however, both of these methods necessitate considerable effort and manual skill [35]. The adjunctive use of regional antibiotics may present an alternative therapeutic approach for oral infectious diseases. However, bacterial biofilm is difficult to eradicate in vivo with minimal antibiotic concentrations [36].

Furthermore, excessive antibiotic dosages may promote bacterial drug resistance and increase biofilm tolerance to antibiotics. Because of these limitations, developing new alternative strategies is urgent [37]. A novel approach that has emerged in recent years is the utilization of nanomaterials possessing enzyme-like characteristics to generate reactive oxygen species (ROS) in situ eliminating microorganisms [38]. The NZs utilized in dentistry research and application primarily catalyze POD, OXD, SOD, and catalase (CAT)-like activities. These activities have the potential to induce irreversible bacterial and biofilm destruction. Because DNA or ions can substantially increase the enzymatic activity of NZs, they can monitor ions effectively [32]. NZs have significantly advanced research in the fields of periodontics and implantology, specifically about the maintenance of periodontal health and the enhancement of implant success rates. We examine NZs for antimicrobial therapy, anti-inflammatory therapy, promotion of tissue regeneration, and synergistic effects in periodontal and peri-implant diseases to illustrate this development [39–41].

Plaque accumulation undoubtedly results in gingival inflammation; its elimination, however, induces a decrease in inflammation. Consequently, patient-assisted plaque eradication is an essential component of non-surgical treatment. This includes interdental cleansing, chemical plaque control, and teeth flossing [42]. Although ultrasonic scaling is a viable initial method for plaque removal, it is inconvenient and necessitates medical intervention. Furthermore, it is imperative to employ a laser of a suitable wavelength that can effectively eliminate calculus while preventing thermal injury to the dentin or structure of the tooth. The ablated surface must be conducive to the reattachment of the soft tissue. Because the wavelength of carbon dioxide lasers is readily absorbed by water, they are suitable for soft tissue surgery. However, because they cause severe thermal injury, they are unsuitable for calculus removal and root surface modification. Furthermore, laser debridement of the root surface is still in its nascent stages. Comparing the numerous studies regarding the protocols and types of lasers employed presents a challenge. However, specific lasers can eliminate calculus and plaque to an extent comparable to that achieved with hand or ultrasonic instrumentation. Nevertheless, they have a documented record of notable adverse effects, most prominently thermal injury to the surface of the roots. Given their relatively high cost, there appears to be a shortage of evidence supporting their use at this time [42, 43].

Furthermore, there is no anti-biofilm effectiveness in anti-demineralization materials presently on the market, such as fluorides, resins, and ceramics. Instead of using invasive restorative treatment, dental nanomaterials, such as nanocatalysts, are being developed to address these issues and react to local environmental stimuli and physiological changes to prevent dental caries [44]. Scientists have made noteworthy advancements in creating innovative, reliable, and effective oral antibacterial medications that induce enzyme activity. In root canals, NZs aid in the prevention of biofilm infection. According to the research by Koo, biofilm plaque can be efficiently eliminated from the surface of dentinal tubules and root canals by activating H_2_O_2_ [45].

Furthermore, the vast majority of microbial infectious diseases are effectively treatable with the extraordinary variety of antimicrobials that are presently accessible. Nevertheless, significant global health challenges include antimicrobial resistance (AMR), adverse effects, and the excessive expense associated with antimicrobials. There is a growing trend of antibiotic resistance among Gram-positive and Gram-negative bacteria that causes infections in hospitals and communities. For instance, nearly forty percent of hospital-acquired *Staphylococcus aureus (S. aureus)* strains are now vancomycin and methicillin-resistant. Moreover, AMR could affect approximately 230 million people annually by 2050 and cumulatively cost the global economy $100 trillion between 2014 and 2050. At present, an estimated 700,000 individuals succumb to fatal infections caused by AMR; this figure is projected to escalate to 10 million by 2050 [46, 47]. The enzymatic breakdown of bacterial cell walls or biofilms, which results in bacterial death, is often described as the antibacterial mechanism of enzybiotics. However, most natural enzymes are unstable during industrial manufacture, which restricts their large-scale use and raises prices. Although further modification and immobilization may somewhat enhance the stability of natural enzymes, they also increase manufacturing costs and operational complexity. When compared to natural enzymes, NZs are a kind of nanomaterial that exhibits enzyme-like activity and are more affordable and stable. Currently, by imitating the enzyme-like properties of natural enzymes, NZ-based nanozybiotics have shown excellent antibacterial application prospects against resistant bacteria. Combining their enzyme-like characteristics with other physiochemical features of NZs, such as PTT and PDT, might enhance their antibacterial efficacy even further. There is still a long way to go until antibacterial tests are clinically transformed since most of them were validated in vitro or topically given using in vivo models. Therefore, it is crucial to investigate novel biocompatible nanozybiotics that use enzyme-like NZs with various antibacterial properties and relevant situations. Researchers think the class of antibiotic substitutes known as nanozybiotics, which are based on NZs with enzyme-like action, is novel [48].

Additionally, CAT, glutathione peroxidase-like (GPx), and superoxide dismutase (SOD) are NZs exhibiting antioxidant properties that demonstrate the potential in mitigating inflammation. As an illustration, scientists utilize mesoporous silica (MSN@Ce) laden with ceria oxide and modify it with polyethylene glycol (MSN@Ce@PEG) to enhance dispersion and biocompatibility. This enabled periodontal ligament stem cells (PDLSCs) to modulate ROS within the cells and promote osteogenic development, thereby protecting them from oxidative stress caused by periodontitis [49, 50].

Before highlighting the significant contribution of NZs to dental health, an overview of the overall research progress of multifunctional NZs in the treatment of oral-related diseases such as periodontitis and peri-implantitis, dental caries, dental pulp diseases, oral ulcers, and periodontal and peri-implant diseases is provided. Furthermore, we discuss the outstanding obstacles that remain in the realm of NZ research and application, as well as anticipate forthcoming issues. We are confident that in the future, novel catalytic nanomaterials will have a significant impact on dentistry.

## Classifications of antibacterial nanozymes

**Fig. 1:**
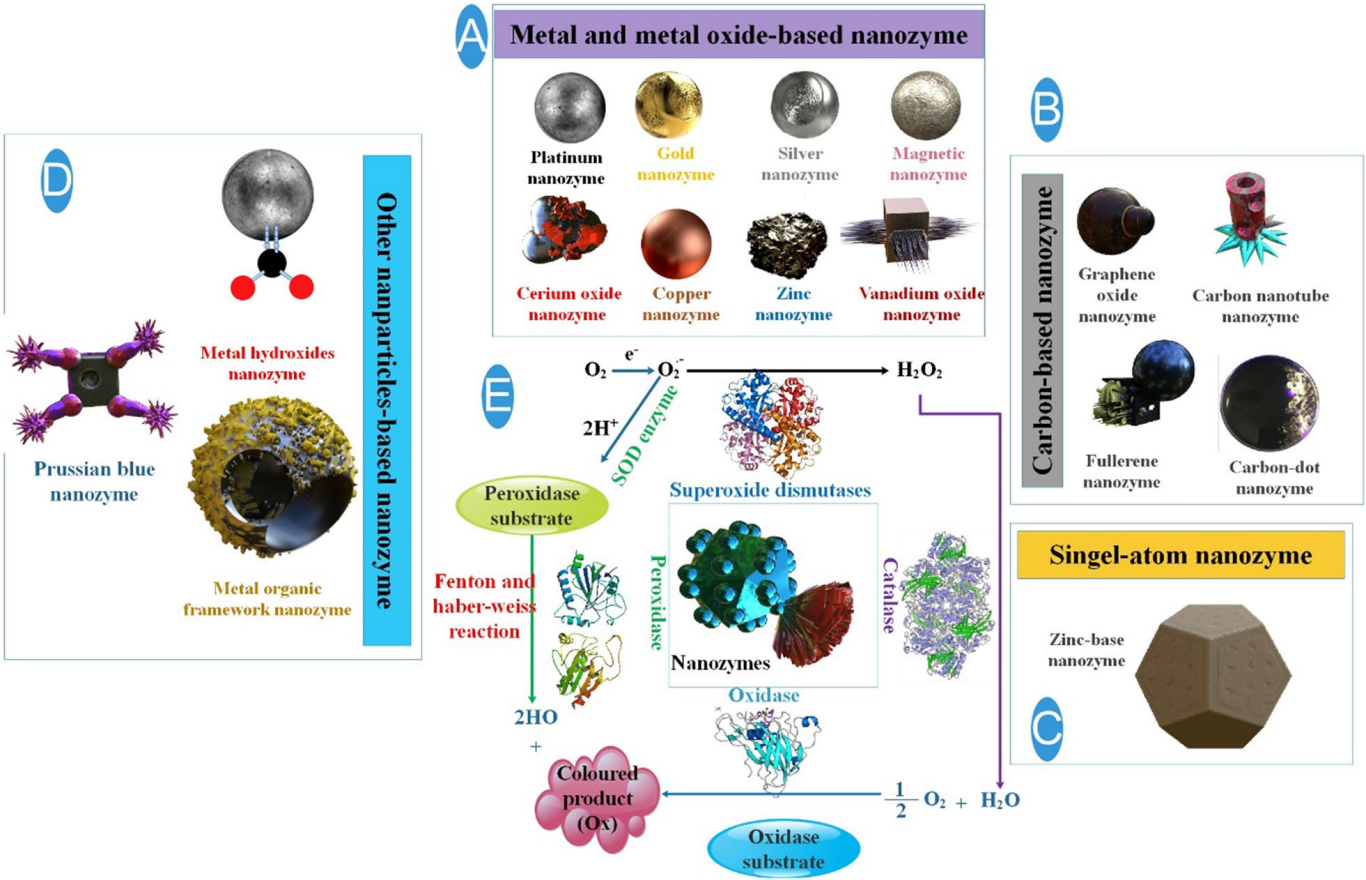
The classification and functions of nanozymes (NZs), including (**A**) metal and metal-oxide NZs (**B**) carbon-based NZs (**C**) single-atom NZs (**D**) metal organic framework-based NZs, prussian blue NZs. **E** To facilitate comprehension in this section, NZs are divided into two categories: (1) oxidoreductase family, which consists of enzymes such as nitrate reductase, oxidase, peroxidase, CAT, and superoxide dismutase; and (2) hydrolase family, which comprises the following enzymes: nuclease, esterase, phosphatase, protease, and silicatein [17]

Classifications and modes of action for NZs Numerous nanomaterials that operate like enzymes have been discovered up to this point. The constituents of NZs typically consist of metal oxide NPs, noble metal nanomaterials, and other materials that primarily display four primary catalytic properties: SOD, CAT, OXD, and POD. Through various techniques, scientists have improved their catalytic qualities, allowing them to selectively and effectively react with specific target molecules [51]. To date, considerable effort has been devoted to the development of antibacterial NZs, which primarily consist of carbon-based nanomaterials, transition metal dichalcogenides/peroxides/oxides, single-atom nanozymes (SAzymes), and metal–organic frameworks (MOFs)-based compounds [21, 52, 53] (Fig. 1).

### Metal-based nanozymes

Several NZs composed of noble metals (e.g., platinum, Ag, and gold (Au)) exhibit significant catalytic activity. Mercaptopyrimidine-conjugated Au nanoclusters (NCs) were developed by Zheng et al. to target resistant superbugs both in vitro and in vivo. The simple adhesion of the NZs to the bacterial surface and subsequent disruption of the cell membrane were facilitated by their positive charge. The induction of intracellular ROS production in bacterial cells was primarily ascribed to intrinsic OXD-like and POD-like activity, which promoted wound healing and killed approximately 99% of bacteria [54]. In addition to its antibacterial efficacy, Zhang et al. assessed the POD-like and ferroxidase-like properties of bimetallic platinum–copper (PtCu) alloy NPs in a mildly acidic media, as well as the ability to detect Fe^2+^. Similarly, Cai et al. created morphology-dependent bactericidal activity in core–shell Pd@Ir bimetallic nanostructures by seed-mediated development. According to this research, the Pd@Ir octahedron’s increased OXD-like activity explained its superior antibacterial activity above Pd@Ir cubes. The Vmax value for the oxidation of 3,3′,5,5′-tetramethylbenzidine catalyzed by Pd@Ir nanocubes was 1.7 times greater, and for the Pd@Ir nano octahedron, it was 4.4 times greater than when catalyzed by Pd cubes alone. Furthermore, it was disclosed in the study that the OXD-like activity of Pd@Ir exhibited an increase when exposed to naturally occurring organic matter. The NZ, when in contact with humic acid (HA), induced significant levels of ROS and facilitated the internalization of the nanostructure by cells [55].

### Metal oxide/sulfide-based nanozymes

A classic example of a biological catalyst, cerium oxide (CeO_2_) NPs possess high POD-like activity due to a reversible redox transition between Ce^4^ + and Ce^3^ + ions. The CeO–H_2_O_2_ system promotes ROS more readily due to its exceptionally high and efficient POD-like activity. Multiple enzymatic activities, including those of SOD, CAT, POD, and OXD, are induced by the surface-rich oxygen (O_2_) vacancies, smooth O_2_ diffusion, and high redox potential of nanoceria of varying sizes and morphologies. Luo et al. created an electrospun nanofibrous membrane (PIL-Ce) composed of imidazolium-type poly (ionic liquid) (PIL)/cerium (IV) ions. In an MRSA-infected mouse model, PIL-Ce demonstrated DNase mimetic catalytic activity and accelerated wound healing. To prevent the spread of drug resistance, the disintegration of resistant genes and the high antibacterial potential of PIL-Ce were both investigated [56]. Since ferromagnetic (Fe_3_O_4_) NPs were first shown to exhibit enzyme-like activity in 2007, much research has been done on NZs, including in-depth analyses of a wide range of NZs and the rapid development of associated nanotechnologies. NZs have opened up new avenues for clinical care, food safety, environmental monitoring, and chemical synthesis as viable substitutes for natural enzymes [57].

For example, fungal infections are considered the largest threat to the global health of all microorganisms, according to the World Health Organization. Enhancing antifungal effectiveness at the infection site while avoiding medication resistance, fungal spread, and off-target effects remains a challenging challenge. In this work, Jun Oh et al. describe a microrobotic platform based on NZs that allows for targeted fungal eradication by accurately guiding localized catalysis to the infection location at the microscale. Dynamic shape transformation and precisely tunable catalytic activation may be achieved in structured iron oxide NZ assemblies via fine-scale spatiotemporal control and electromagnetic field frequency modulation. Motion, velocity, and shape all have an impact on catalytic activity, which makes it possible to control the generation of ROS. Surprisingly, fungal cell surfaces (*Candida albicans*) are addressed by NZ assemblies, which allow targeted ROS-mediated destruction in situ and concentrated accumulation. Using these tunable characteristics and selective binding to fungus, localized antifungal efficacy is achieved using in vivo-like cell spheroid and animal tissue infection models. Structured NZ assemblies are guided toward *Candida albicans* infection sites using programmable algorithms. This allows accurate directed spatial targeting and on-site catalysis, both of which help to eradicate the fungus quickly. Using this NZ-based microrobotics method, pathogens may be eliminated at the site of infection using a highly targeted and effective treatment strategy [58]. Robots powered by magnetism can perform intricate tasks in biological settings with little harm. However, robots designed to injure detrimental biostructures might potentially have a significant impact. In light of the approaching antibiotic age, innovative methods for eliminating bacterial biofilms are crucial. Biofilms are enduring, tightly adherent forms often connected to the emergence of illnesses resistant to drugs and the deterioration of surfaces. Reinfection results from existing therapies' insufficient ability to eradicate microorganisms. In a study, researchers created catalytic antimicrobial robots (CARs) that have remarkable effectiveness and control in the removal, degradation, and elimination of biofilms. Iron oxide NPs, which have both catalytic and magnetic capabilities, are used in CARs. These NPs: (i) generate bactericidal free radicals; (ii) break down the biofilm's exopolysaccharide (EPS) matrix; and (iii) use magnetic field-driven autonomous assemblies to remove the scattered biofilm debris. Researchers develop two distinct CAR systems. The first platform, the biohybrid CAR, is made of NPs and waste products from the breakdown of biofilms. Magnetic field gradients assemble NPs and the biodegraded products into a plow-like superstructure after EPS rupture and catalytic bacterial death. The biohybrid CAR effectively and selectively removes biomass when exposed to an external magnetic field, which prevents biofilm from regenerating. To accomplish targeted elimination with microscale precision, biohybrid CARs may rove along predefined trajectories or cover large surface regions. The second platform, the 3D molded CAR, is a flexible robotic polymer with integrated catalytic-magnetic NPs. It is created in a specially made 3D printed mold and intended to carry out certain functions inside limited areas. Vane-shaped CARs remove biofilms from the curved walls of cylindrical tubing, whereas helicoid-shaped CARs break through biofilm obstructions and kill bacteria. Moreover, researchers demonstrate how CARs may be applied to anatomical areas that are very limited within the human dentition. 'Kill-degrade-and-remove' CARs systems have the potential to significantly reduce biofouling on a variety of surfaces and medical equipment as well as tackle chronic biofilm infections [59].

### Carbon-based nanozymes

The biomedical field has extensively used carbon-based nanomaterials, including CDs, CNTs, graphene and its derivatives, carbon nitride, and fullerene, due to their biocompatibility, physiochemical properties, and ability to mimic multiple enzymes. In a broad pH range, Wang et al. synthesized a series of CNTs (o-CNTs) that were abundant in oxidized groups and exhibited superior POD-like activity. The active catalytic centers on the surface of o-CNTs were the carbonyl group, while the competitive sites were the carboxyl and hydroxyl groups [60]. The carboxyl group has a stronger inhibitory tendency on the catalytic propensity than the hydroxyl group because of its intrinsic negative charge and propensity to create hydrogen bonds. Consequently, o-CNTs-BrPE, or 2-bromo-1-phenylethanone-modified o-CNTs, were made to lessen the carboxyl group in the NZ's inhibitory function. o-CNTs-BrPE demonstrated strong POD-like action as the number of competing sites dropped, allowing catalysis of H_2_O_2_ to ·OH and eliminating bacteria and shielding tissues from purulent inflammation and edema brought on by bacteria [56, 61].

### Metal–organic frameworks (MOFs)

MOFs are distinct crystalline and porous nano/microstructures produced by organic linkers with two or more coordinating positions bridging metallic nodes of single metal ions or clusters of few metal ions. A novel porous coordinating polymer structure is attributed to the labile bonds between organic linkers and metal ions or clusters. MOFs exhibit remarkable mechanical and chemical properties. When juxtaposed with traditional NZs, MOFs-based NZs possess several significant benefits: (i) The wide array of active metal ions/clusters and organic linkers at one's disposal provides opportunities for the development of numerous NZs that possess inherent and modifiable enzyme-like characteristics [22]. (ii) Exposed active catalytic sites with improved enzyme-mimicking qualities are provided by the metal nodes and organic linkers systematically placed in an architectural framework. (iii) Natural enzymes for various cascade systems may be accommodated by the porous structure with nano/micropores, which can also promote high substrate channeling to the active sites. (iv) MOFs’ adaptable porosity and varied forms allow for effective size control of reacting molecules, enhancing catalytic activity with a high degree of substrate selectivity [62]. (v) MOFs with high specific surface area have a varied geometric structure that provides a tunable platform for further modification, which enhances their catalytic activity by adding new features. Because of these benefits, a lot of work has been put into creating MOFs-based NZs for biological catalysis and sensing in recent years. MOF-based NZs have shown remarkable potential in the biomedical domain owing to their adaptable architectures, high activity, and strong stability. Interestingly, the investigation of MOFs with enzyme-mimicry characteristics for bacterial theranostics applications was aided by the rapid creation of catalytic nanomedicines. For the creation of innovative NZs with hereditary catalytic activity for the treatment and diagnosis of bacterial infections, MOF is thought to be a viable platform [63].

Because H_2_O_2_ is a prevalent ROS, it has been utilized extensively to combat pathogenic bacterial infections. However, excessive H_2_O_2_ can cause harm to healthy tissues and impede the healing process. POD-like nanomaterials exhibit great potential as NZs in this context due to their ability to enhance the antibacterial activity of H_2_O_2_ without introducing the toxicity associated with high concentrations of H_2_O_2_. Using in situ reduction, ultrasmall Au NPs (UsAuNPs) are produced on ultrathin 2D MOFs in this study. Combining the benefits of UsAuNPs and ultrathin 2D MOFs, the UsAuNPs/MOFs hybrid exhibits an exceptional POD-like activity in decomposing H_2_O_2_ into toxic hydroxyl radicals (·OH). The UsAuNPs/MOFs NZ, in its as-prepared state, demonstrates remarkable antibacterial efficacy against *S. aureus* and *Escherichia coli* (*E. coli*), two Gram-negative and Gram-positive bacteria, respectively, when a minimal amount of H_2_O_2_ is added. This study presents compelling evidence regarding the antibacterial potential of a hybrid NZ and underscores its significant potential for forthcoming clinical implementations [64]. Furthermore, it is noteworthy that while SAzymes containing MOF derivatives have been documented, the utilization of single-atom dopped MOF as NZs in periodontitis treatment has not been documented nor implemented to the best of our knowledge [65].

### Single-atom nanozymes (SAzymes)

NZs, nanomaterials that exhibit enzymatic activity, have been the subject of extensive research. Inadequate substrate selectivity, a complex composition, and a low density of active sites have impeded the maturation and widespread adoption of NZs. A leader in the field of catalysis, enzymes with atomically dispersed active sites are distinguished by their outstanding performance. Researchers highly value the following characteristics of SAzymes: optimal atom utilization rate, economical cost, clearly defined coordination structure, and active sites [66]. SAzyme is the current focal point of NZ research. Its inherent characteristics, including high activity, stability, and affordability, make it an excellent alternative to natural enzymes. Furthermore, compared to conventional NZs, its intrinsic qualities—namely, optimized atom utilization and precisely defined geometric and electronic structures—contribute to its superior catalytic activities and specificity [67]. An instance of this can be seen in the synthesis of alternative Cu SAzymes featuring atomically dispersed Cu sites anchored on ultrathin 2D porous N-doped carbon nanosheets (CuNx-CNS) and tunable N coordination numbers in the CuN_x_ (x = 2 or 4) sites is described by researchers utilizing a Cu and silk fibroin (Cu-SF) complex strategy. The triple POD, CAT, and OXD-like activities of the CuNx-CNS SAzymes enable the conversion of H_2_O_2_ and O_2_ to ROS via parallel POD and OXD-like reactions or cascaded CAT and OXD-like reactions. In contrast to CuN_2_-CNS, increasing the N coordination number from 2 to 4 confers greater multienzyme activities upon the SAzyme (CuN_4_-CNS), attributed to its enhanced electron configuration and reduced energy barrier. In contrast, CuN_x_-CNS exhibits robust absorption in the second near-infrared (NIR-II) biowindow, facilitating photothermal treatment in deep tissues and NIR-II-responsive enhancement of ROS production. The optimal CuN_4_-CNS inhibits multidrug-resistant bacteria and eliminates resistant biofilms, demonstrating high therapeutic efficacy against both superficial skin wounds and deep implant-related biofilm infections, as shown by in vitro and in vivo results [68].

## Nanozymes in antibacterial applications

Living things include enzymes that can change a range of substrates into ROS, used to fight bacterial invasion. Oxidative salivary enzymes, which support the host's natural defense mechanism, prevent or limit the growth of oral pathogens; polysaccharide hydrolases, such as mutanases and dextranases, break down essential carbohydrate components of the biofilm matrix; and proteases alter cell-to-cell interactions or prevent bacteria from adhering to oral surfaces [69]. Bacteriostatic hypothiocyanite (OSCN-), a mild oxidizing agent, is generated through the enzymatic catalysis of thiocyanate ion (SCN-) oxidation by hydrogen peroxide (H_2_O_2_) by the enzyme lactoperoxidase (LP). This process takes place within secretory fluids. However, H_2_O_2_ retains its antibacterial characteristics without thiocyanate (SCN-) and LP. Consequently, LP has the potential to either protect bacteria from the harmful effects of H_2_O_2_ by transforming it into a less potent oxidizing agent or it can enhance antibacterial efficacy by utilizing H_2_O_2_ to produce a more potent growth and metabolism inhibitor for bacteria. An investigation was conducted to determine the function of LP by assessing the antibacterial properties of H_2_O_2_ and the LP-H_2_O_2_-SCN system through the inhibition of bacterial growth and metabolism and the loss of viability. *Streptococci* are protected from H_2_O_2_ mortality by LP and SCN, and elevated concentrations of H_2_O_2_ for protracted periods result in a potent bactericidal effect, according to the findings. As an inhibitor of bacterial growth and metabolism, LP, H_2_O_2_, and SCN-combinate significantly outperform H_2_O_2_ alone [70]. *Streptococcus sanguinis* (*S. sanguinis*) is a strain of Gram-positive bacteria that causes dental caries. Creating new antibacterial agents is crucial as many antibacterial agents are resistant to microorganisms. Among the enzymes that support the cell wall is the enzyme murA. The first stage of peptidoglycan biosynthesis, which includes the creation of the cell wall, is catalyzed by MurA. By suppressing MurA, the germs may be eliminated with effectiveness and efficiency. Medicinal plants and other natural items include bioactive chemicals and antibacterial agents. According to reports, *Piper betle* L. possesses potent antibacterial properties. A minimum inhibitory concentration (MIC) and maximum barrier concentration (MBC) of 39.1 and 78.1 μg/mL, respectively, were the results of the antibacterial compound allylpyrocatechol’s inhibitory activity against *S. sanguinis* at a concentration of 1%. An inhibition zone of 11.85 mm was also observed. Two allylpyrocatechol derivatives, which were found to be more powerful than the reference molecule fosfomycin and had binding activities of − 5.4 and − 4.6%, respectively, were used to anticipate the molecular inhibitory mechanism of allylpyrocatechols against MurA [71]. However, several inherent drawbacks, such as their high cost, low stability, and restricted capacity for production scaling, significantly impede their continued use as antibacterial agents. Therefore, it is necessary to investigate effective antibacterial drugs at clinical translations [72]. Upon interaction with a bacterium, engineered NPs have the potential to induce ROS, discharge heavy metals, impair proton efflux pumps, disrupt electron transport chains, and rupture cell membranes. One such strategy is ROS, which has demonstrated rapidity, efficacy, and broad-spectrum activity against bacteria and cancer, and notably, does not appear to promote the development of drug-resistant microorganisms. Additionally, ROS reacts with the DNA and lipids of latent bacteria, specifically “superbugs” and recalcitrant biofilms, and possesses potent antibacterial properties [73].

The advent of nanotechnology has facilitated the creation of NZs, which offer a potential therapeutic approach for bacterial infections. It is widely recognized that the antibacterial mechanism of NZs can be broadly classified into the subsequent categories: By converting the corresponding substrate H_2_O_2_ or O_2_ into ROS such as ·OH or singlet oxygen (^1^O_2_), POD or oxidase mimics are capable of producing an antibacterial effect. Furthermore, prodrugs can be converted to antibiotics in the presence of NZs via bio-orthogonal techniques, producing drugs with an antibacterial adequate impact. Moreover, the phospholipid structure of bacterial cell membranes will be decomposed due to the phosphatase-like activity of NZs, resulting in bacterial mortality. The first antibacterial pathway has received the most research to date. Although numerous researchers have investigated NZs for antibacterial purposes, most NZ-based systems lack targeting capabilities. As a result, complications regarding the adverse effects and therapeutic efficacy may arise. In light of these considerations, several research studies have modified small molecular groups on the surface of NZs to target bacteria. The NZ-based targeted antibacterial system consists primarily of aptamers and particular small molecules, including mannose, C18-PEGn-benzeneboronic acid (CPB), dextran, and others. By forming specific bonds with bacteria, these substances are capable of causing bacterial death via the catalytic activity of NZs. This section focuses primarily on the targeting effect of NZs as an antibacterial system application [74].

A prospective alternative for combating microbes, NZs have recently become a research hotspot due to their low cost, high stability, scalability, and multiple functionalities. In contrast to conventional antibiotics, NZs exhibit a reduced propensity to induce bacterial resistance by capitalizing on the advantageous properties of nanomaterials, including favorable membrane permeability and innocuous biocompatibility. In contrast, antibacterial methods based on NZs exhibit distinct advantages compared to alternative antibacterial strategies [75]. Moreover, their catalytic activities can efficiently eliminate bacterial biofilms. Beyond that, the distinctive physicochemical properties of NZs enable them to possess additional functionalities not found in natural enzymes, thereby facilitating catalytic activities modulated by composition, size, and shape. The unique physicochemical characteristics of these substances present an opportunity to create multifunctional antibacterial agents. It is essential and highly recommended to develop novel bactericides that effectively eliminate bacteria without fostering the growth of resistance or causing biosafety concerns. NZs, which are inorganic nanostructures possessing inherent enzymatic activities, have garnered increasing attention from scientists due to their remarkable properties. NZs are more effective than natural enzymes at destroying a wide variety of Gram-positive and Gram-negative bacteria, thereby bridging an essential gap between biology and nanotechnology. NZs, being highly effective nanoantibiotics, exhibit remarkable broad-spectrum antimicrobial characteristics while exhibiting minimal biotoxicity [22].

Additionally, NZs have antibacterial applications. It is essential to eliminate *S. mutans* and the biofilm that forms on the tooth's surface to prevent dental caries. Scholars have developed a collection of NZs capable of operating effectively in acidic pH environments [50]. The utilization of NZs possessing OXD-like and POD-like characteristics to catalyze the conversion of the corresponding substrate to ROS in a physiological setting has the potential to expand the range of applications in the antibacterial field. However, the efficacy of the generated ROS against bacteria is hindered by their short diffusion distance in the environment and their high reactivity; this compromises the biosafety and antibacterial activity of the ROS. Therefore, the secret to achieving the effective antibacterial activity of NZs is the combination of enzyme-like activity and bacterial binding ability [75, 76].

By generating ROS through enzyme-mimetic catalytic reactions, NZs can efficiently and swiftly destroy bacteria, rendering them viable substitutes for antibiotics in antibacterial applications. Despite this, the ability of NZs to eradicate bacterial infections is severely hampered by their inadequate catalytic activity. Enzymes that possess an atomical dispersion of active metal sites have demonstrated exceptional enzyme-like activities and have made significant strides in recent years in the field of antibacterial applications by maximizing atom utilization. Stunningly superior enzyme-like activities have been exhibited by SAzymes as a result of their atomic dispersion of active metal actives and similar atomic configuration to that of natural enzymes; this enables them to generate an abundance of ROS to eliminate bacteria. SAzymes, which are advantageous because they are inexpensive, highly stable, and compatible, have generated considerable interest in antibacterial applications [77]. Dental caries is still the most common illness in humans because of oral biofilms, even with the widespread use of fluoride as the major antibiotic. It has been established that hydrogen peroxide catalytic activation of ferumoxytol (Fer), an iron oxide NP that was recently licensed by the FDA, breaks down and destroys biofilms that cause tooth caries. Conversely, fer has no impact on the demineralization of enamel acid. Researchers established that stannous fluoride (SnF_2_) and ferric chloride exhibit a strong synergy that significantly outperforms each element alone in inhibiting biofilm growth and enamel damage. Surprisingly, adding Fer to aqueous solutions improves SnF_2_'s stability while simultaneously increasing Fer’s catalytic activity naturally and without the need for additions. Notably, even at four times lower concentrations, the combination of SnF_2_ and Fer shows significant effectiveness against dental caries in vivo without adversely affecting the oral microbiota or host tissues. The results of this study show that authorized medications and SnF_2_ stabilization have a solid therapeutic synergy that may be used to lower fluoride exposure and prevent widespread oral illnesses [78].

## Application of nanozymes in oral antibacterial treatment

Bacterial infection remains an escalating concern in global health, where antibiotics remain the most widely acknowledged treatment paradigms [79]. Nevertheless, the misuse and overuse of antibiotics have resulted in a surge in multidrug resistance, which has adversely affected therapeutic efficacy and contributed to elevated mortality rates [80]. Moreover, the propensity of bacteria to establish biofilms on both living and nonliving surfaces exacerbates the challenge of combating bacteria, as the extracellular matrix can serve as a formidable barrier to environmental stress and antibiotic penetration [81]. The failure to eradicate microbes and biofilms frequently results in the development of persistent infections, malfunctioning implants, and harm to the device. Hence, the development of alternative antimicrobial agents that prevent the emergence of bacterial resistance is critical [82]. By studying the mechanisms by which natural enzymes disrupt metabolism, such as quorum sensing, programmed death, and cellular structural integrity, artificial enzymes that imitate the functions of these enzymes will offer unparalleled prospects for the fight against bacteria [83]. Furthermore, unlike natural enzymes, synthetic enzymes exhibit significantly enhanced resistance to extreme conditions, catalytic activity that is more easily modifiable, and the capability to be produced on a large scale for practical applications [19].

A multitude of NZ-assisted approaches have been successfully developed thus far to serve as theranostics for various diseases. These approaches capitalize on the low cost, high stability, and multienzyme-like properties of NZs [50]. Oral infection, being the most prevalent oral disease, presents a worldwide threat to human health, and the available therapeutic alternatives are inadequate to address all the clinical complications. NZs, by their remarkable efficacy, can be routinely utilized in the detection and management of a multitude of oral infectious diseases [84]. Even more significantly, NZs can have their shape, size, and composition modified, which confers an extensive array of enzymatic and antibacterial properties. Metal-based compounds, carbon-derived nanomaterials, transition metal dichalcogenides, peroxides, oxides, SA enzymes, and MOFs have all been employed in antibacterial research [85]. Firmicutes, Bacteroidetes, Proteobacteria, Actinobacteria, Spirochaetes, and Fusobacteria predominated in the oral bacterial community. Among these, *Streptococcus mutans* (*S. mutans*) and *Lactobacillus* have been the subject of extensive research and are regarded as specific caries pathogens. Periodontitis has been linked to several microorganisms, including *Porphyromonas gingivalis, Treponema denticola*, and *Tannerella forsythia* [86]. Furthermore, the antibacterial activity of NZs is predominantly mediated by the catalytic processes of POD and OXD, which convert H_2_O_2_ into ·OH to control ROS. ROS are a class of small molecules that the host's phagocytes can produce; examples include ·OH, superoxide radicals, and H_2_O_2_ [87]. The generation of ROS by negatively charged metal NPs upon interaction with positively charged bacterial cell wall surfaces has exhibited antibiotic-like properties across a range of disorders [88]. Several NZs exhibit distinct benefits in oral antibacterial therapy, according to another study, even though their active mechanisms have not been exhaustively investigated and comprehended. A synopsis of the primary procedure follows: After brief local exposure, NZs remain within the biofilm structure of three-dimensional (3D) dental plaque, and H_2_O_2_ swiftly converts to free radicals at acidic PH to degrade EPS and eliminate bacteria [89–91].

NZs play three preeminent functions in this procedure. (1) Adequate bioavailability requires that the substance remains in the plaque biofilm and maintains its activity; (2) Stable in physiological environments but activated in a PH-dependent manner in specific acidic pathogenic microenvironments produced by plaque biofilms. For instance, Fe_3_O_4_ NPs possessing POD-like activity catalyze H_2_O_2_ exclusively after penetrating the plaque biofilm. (3) Mitigate the detrimental effects on healthy tissues induced by comparatively high concentrations of H_2_O_2_ (0.5–3%) commonly employed in conventional antibacterial methods. By converting H_2_O_2_ to free radicals, POD-like NZs are capable of generating an exceptional antibacterial effect, reducing the concentration of H_2_O_2_ used for antibacterial purposes significantly, and enhancing biological safety. Furthermore, oral antibacterial applications encompass the following: prevention of peri-implantitis, treatment of dental caries and pulp disease, and treatment of oral ulcers [32].

**Fig. 2:**
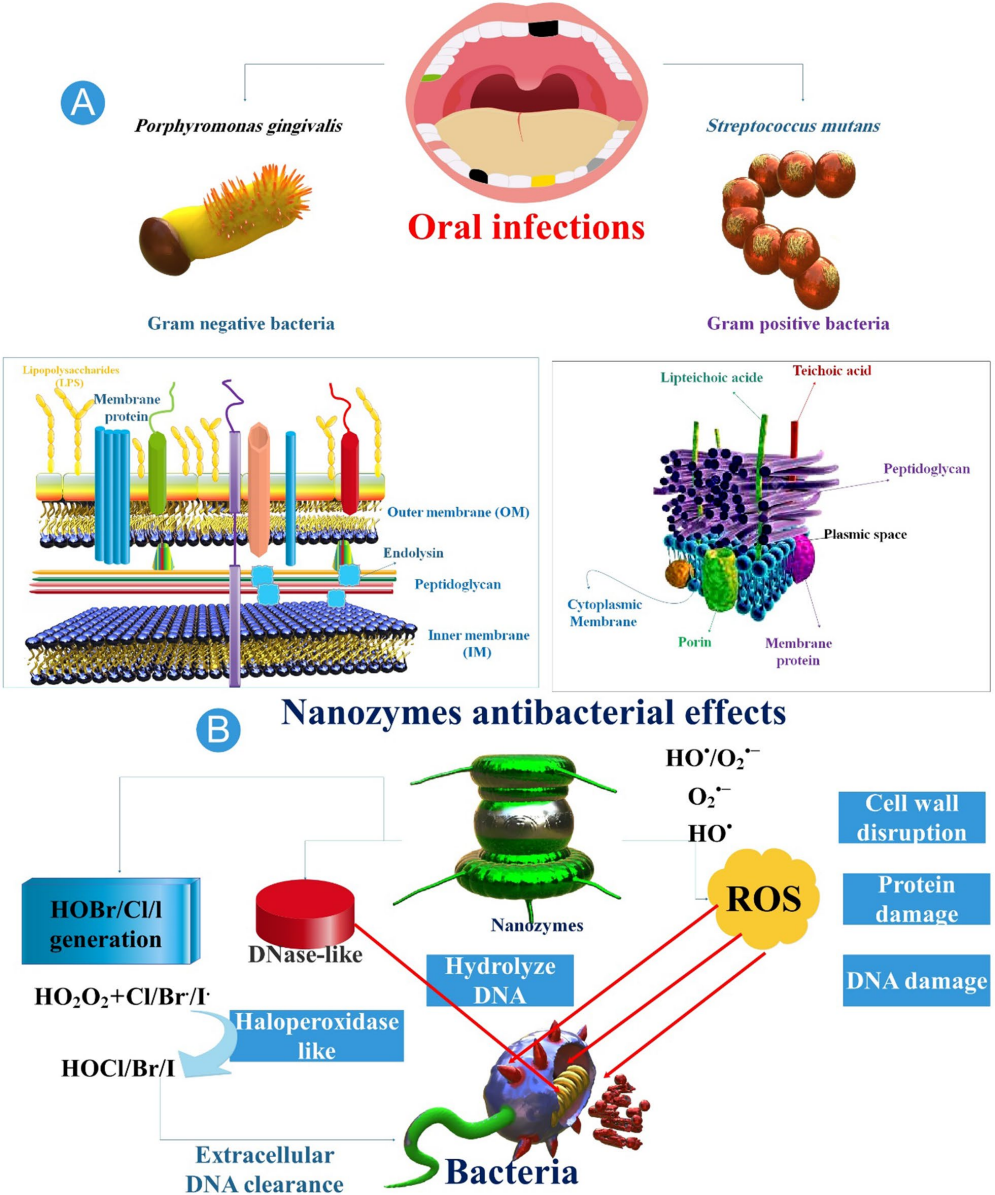
The antibacterial processes and properties of nanozymes (NZs). **A** *Streptococcus mutans* and *Lactobacillus* are the two most prevalent species of bacteria that cause dental cavities. Gum disease may result from the growth of *Treponema denticola* and *Porphyromonas gingivalis*, which can make your gums swell. **B** As we all know, the antibacterial mechanism of NZ is mainly divided into the following categories: POD or oxidase mimics can transfer corresponding substrate H_2_O_2_ or oxygen (O_2_) into ROS such as ·OH or singlet oxygen (^1^O_2_), thereby achieving antibacterial effect [94]

The U.S. Food and Drug Administration has approved Fer, an NP formulation, for systemic administration to treat iron deficiency. Furthermore, researchers demonstrated that Fer inhibits tooth decay (dental caries) and disrupts intractable oral biofilms via intrinsic POD-like activity. Fer forms a complex with the ultrastructure of biofilms and produces free radicals from H_2_O_2_, which induce in situ bacterial mortality through the disruption of cell membranes and degradation of extracellular polymeric substance matrices. When combined with modest concentrations of H_2_O_2_, Fer prevents acid injury to the mineralized tissue and inhibits biofilm accumulation on natural teeth in an ex vivo biofilm model derived from humans. In a rodent model of the disease, topical oral treatment with Fer and H_2_O_2_ inhibits the development of dental caries in vivo, thereby averting the initiation of severe tooth decay (cavities). Gingival and mucosal tissues, as well as the diversity of oral microbiota, are not negatively impacted, according to histological and microbiome analyses. Investigators' findings demonstrate that Fer has a novel biomedical application as a topical treatment for a common and expensive oral disease caused by biofilm. Additionally, our group investigated topical Fer as a NZ to prevent dental caries (tooth decay) by killing bacteria and disrupting biofilm. In this experimental setup, 1% H_2_O_2_ exposure was followed by topical administration of Fer at a concentration of 1 mg/ml in the oral cavity, which served as a rodent model of dental caries. In a recent study, researchers utilized an analogous topical treatment protocol to specifically target biofilms that are accountable for tooth caries in the human oral cavity [90, 92, 93] (Table 1) (Fig. 2).

**Table 1:** Nanozyme in oral bacterial infections

Nanozymes	Oral disease	Antibacterial mechanism and function	Reference
Ferumoxytol (Fer)	Tooth decay	Researchers Investigated the use of topical Fer as a nanozyme to prevent dental caries (tooth decay) by killing bacteria and disrupting biofilm	[90, 92, 93]
Dex-NZM	Severe caries	Researchers presented dextran-coated iron oxide nanoparticles (Dex-NZM) that exhibit potent catalytic (POD-like) activity at acidic pH levels, selectively target biofilms to prevent severe caries, and do so in vivo without affecting adjacent oral tissues	[95]
Glucose-OXD	Dental caries	Utilize a nanohybrid system to increase intrinsic H_2_O_2_ production and induce pH-dependent ROS generation to effectively target biofilm virulence under pathological (sugar-rich/acidic)	[96]
Iron oxide nanozymes or iron sulfide nanozymes	Dental caries	Integrating H_2_O_2_-producing bacteria and FeSN could potentially offer a novel approach to eradicating oral biofilms during dental caries therapy	[98]

**Fig. 3:**
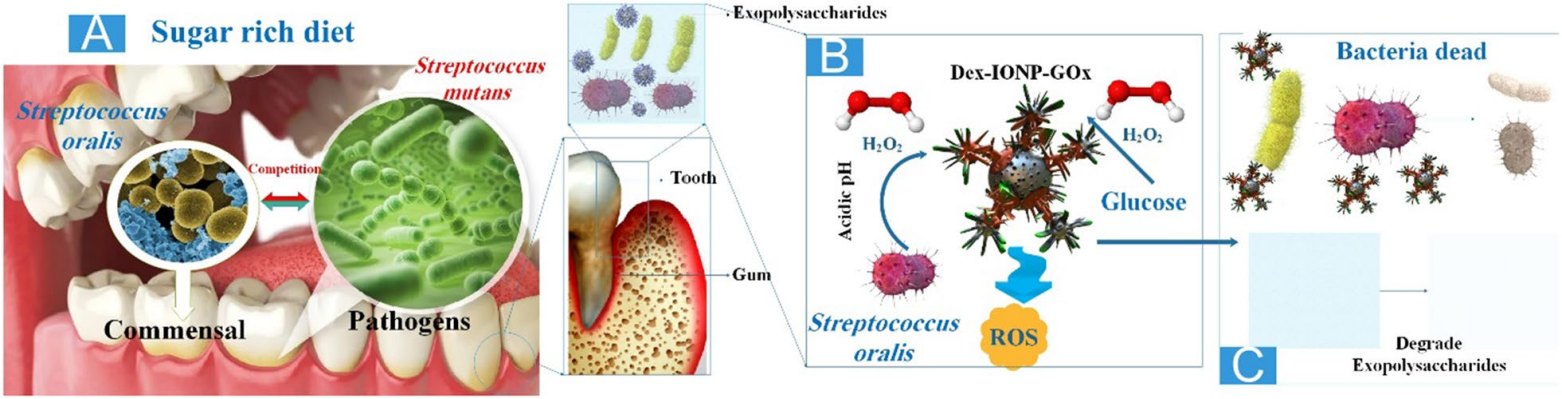
**A** The microbial balance between commensals and pathogens can be disrupted by frequent sugar consumption and poor oral hygiene. **B** Dex-IONP was supplemented with glucose oxidase (GOx) by Koo's group to produce the Dex-IONP-GOx bifunctional nanohybrid system. GOx can convert glucose into H_2_O_2_ within the plaque biofilm, thereby depriving *S. mutans* of its food source. Without additional H_2_O_2_, Dex-IONP can directly catalyze H_2_O_2_ in an acidic microenvironment and generate ROS to destroy microorganisms. **C** Their findings indicate that the efficacy of this system surpasses that of Dex-IONP alone by a substantial margin. Moreover, it exhibits a more precise targeting of *S. mutans* (> 107 reduction) while displaying minimal impact on other symbiotic beneficial bacteria [96, 97]

Dental caries can be induced by acidogenic oral biofilms, which demineralize the enamel-apatite present on teeth. At present, antimicrobial agents exhibit limited effectiveness and fail to target the protective matrix or acidic pH present in biofilms. It was recently demonstrated that catalytic NPs could disrupt biofilms, but they lacked the stabilizing coating for clinical applications. In this study, researchers presented dextran-coated iron oxide nanoparticles (Dex-NZM) that exhibit potent catalytic (POD-like) activity at acidic pH levels, selectively target biofilms to prevent severe caries, and do so in vivo without affecting adjacent oral tissues. NP formulations with dextran coatings (ranging in molecular weight from 1.5 to 40 kDa) were produced and evaluated for their bioactivity and catalytic performance. The optimal dextran coating for catalytic activity, biofilm assimilation, and antibiofilm properties was determined to be 10 kDa. The catalyst activity is attributed to the presence of iron oxide centers, according to mechanistic investigations. Stability is maintained by the dextran on the NP surface, which does not impede catalysis. Coating NZM with dextran enabled its incorporation into the structure of EPS and its binding to biofilms; this interaction triggered the production of H_2_O_2_ to destroy bacteria locally and degrade the EPS matrix. Unexpectedly, dextran coating prevented gingival cell binding while increasing selectivity toward biofilms. In addition, treatment with Dex-NZM/H_2_O_2_ significantly decreased the initiation and severity of caries lesions (in vivo, without affecting gingival tissues or oral microbiota diversity) compared to the control group or treatment with Dex-NZM or H_2_O_2_ alone. Hence, dextran-coated NZs exhibit promise as a viable alternative therapeutic approach for managing dental caries and potentially other diseases associated with biofilm [95] (Fig. 3).

Commensal bacteria aid in regulating opportunistic pathogens by producing bioactive byproducts like H_2_O_2_. However, excessive sugar intake disrupts homeostasis and encourages the accumulation of pathogens in acidic biofilms, which are responsible for tooth caries. In this study, scientists utilize a nanohybrid system to increase intrinsic H_2_O_2_ production and induce pH-dependent ROS generation to effectively target biofilm virulence under pathological (sugar-rich/acidic) conditions. The nanohybrid material comprises glucose-OXD, which facilitates the conversion of intrinsic H_2_O_2_ to ROS at acidic pH by iron oxide NPs with POD-like activity. Significantly, it eliminates *S. mutans* (the pathogen) while leaving *Streptococcus oralis* (the commensal) unaffected using preferential pathogen-binding and in situ ROS production. In addition, in a rodent model, nanohybrid interventions significantly diminished dental caries. In contrast to chlorhexidine, which disrupted the diversity of oral microbiota as the positive control, the nanohybrid exhibited a considerably greater efficacy while modulating microbial activity associated with dental health in vivo, without affecting soft-tissues or the oral-gastrointestinal microbiomes. The therapeutic specificity of a bifunctional hybrid NZ against a biofilm-associated disease was demonstrated by the data in a controlled fashion when activated under pathological conditions [96].

A methodology is presented by researchers wherein NZs possessing POD-like activity are integrated with bacteria producing biogenic H_2_O_2_ to eradicate oral biofilms in the context of caries treatment. To replicate the oral environment, we examine the impact of iron oxide NZs or iron sulfide NZs on an *S. mutans* biofilm in the presence of H_2_O_2_-producing *S. gordonii* using a saliva-coated hydroxyapatite disc and a sectioned human tooth. The results of bacterial viability assays and biofilm morphology characterization indicate that the co-administration of NZs and bacteria results in a significant reduction of both the bacterial population (5 lg) and the biofilm matrix (85%). Hence, integrating H_2_O_2_-producing bacteria and iron-based nanozymes (FeSN) could potentially offer a novel approach to eradicating oral biofilms during dental caries therapy [98].

## Nanozyme in the treatment of dental diseases

The escalating global incidence of periodontal and peri-implant diseases has garnered considerable interest. NZs, which possess enzyme-like activity and are multifunctional nanomaterials, have established a presence within the biomedical domain. NZs have made significant contributions to plasmonics and implantology research concerning the maintenance of periodontal health and the enhancement of implant success rates [41]. In most cases, oral diseases result from bacterial infection and inflammation. ROS, produced by bacterial infection and autologous inflammation tissue, are crucial to this process. Consequently, eliminating an excess of intracellular ROS may represent a viable anti-inflammatory treatment strategy. In treating inflammation-related diseases, NZs, which can maintain intracellular redox balance and safeguard cells from oxidative damage, have demonstrated promising application prospects due to the accelerated development of nanomedicines [99]. Conventional dental materials exhibit a limited number of inevitable drawbacks that detrimentally impact the efficacy of dental procedures and ultimately result in treatment failure. Dental research investigates the potential of various nanoenzymes to treat periodontitis, caries, and oral ulcers. Based on their anti-inflammatory, antibacterial, and immunomodulatory properties, the enzymes find use. The preponderance of research published within the last two to thirty years has focused on NPs, suggesting that nanotechnology and the characteristics of resources at these dimensions are of immense interest [100]. Plaque dental caries is a prevalent infectious oral disease affecting one billion people globally. Oral biofilm is the source of numerous diseases that pose a threat to oral health and have the potential to progress to systemic conditions, including Alzheimer's disease, diabetes, and atherosclerosis. These conditions entail substantial financial burdens and catastrophic complications. Prominent progress has been achieved by scientists in the development of novel, consistent, and productive oral antibacterial drugs that stimulate enzyme activity. NZs contribute to the prevention of biofilm infection in root canals. Activating H_2_O_2_ can effectively eradicate biofilm plaque from the surface of a root canal and dentinal tubules, according to Koo's research [101–103]. Oral ulcers have been linked in numerous studies to bacterial and viral infections, allergies, deficiencies in vitamins and trace elements, systemic diseases, and genetic susceptibility. Present treatment methods lack unique pharmaceutical agents; therefore, it is necessary to develop therapeutic approaches that boost the immune system and promote ulcer healing. Naha et al. report that the healing of oral ulcers is accelerated by vitamin B_2_-modified Fe_3_O_4_ NZs exhibiting anti-inflammatory and antibacterial properties [104, 105]. As stated by the researchers, this alteration substantially enhanced its SOD-like activity and propensity to scavenge ROS. Research on cellular antioxidation demonstrated that these enzymes exhibited biocompatibility and cellular protection against H_2_O_2_. Killing *S. mutants*, reducing local inflammatory factors, and removing ROS, these NZs accelerate the healing of rodent oral ulcers. This antibacterial mediator resembling an enzyme may represent a viable treatment for oral ulceration [95]. POD, SOD, OXD, and CAT-like activities comprise the majority of the NZs' catalytic activity in dental applications and research. These activities have the potential to induce irreversible bacterial and biofilm annihilation. Given that NZs may exhibit a substantial increase in enzymatic activity upon exposure to DNA or ions, they possess the potential to function as colorimetric biosensors for the detection of oral cancer-associated bacteria, ions, or DNA. NZs can foster the regeneration of both soft and hard tissues by facilitating cell adhesion, proliferation, and differentiation within a sterile milieu. The utilization of NZs in dentistry has demonstrated encouraging outcomes by addressing the limitations of traditional H_2_O_2_ concentrations, mitigating oxidative stress induced by the environment during cellular proliferation and differentiation, eradicating oral flora through biofilm degradation, and rapidly and easily monitoring oral flora and *S. mutants* [100].

**Fig. 4:**
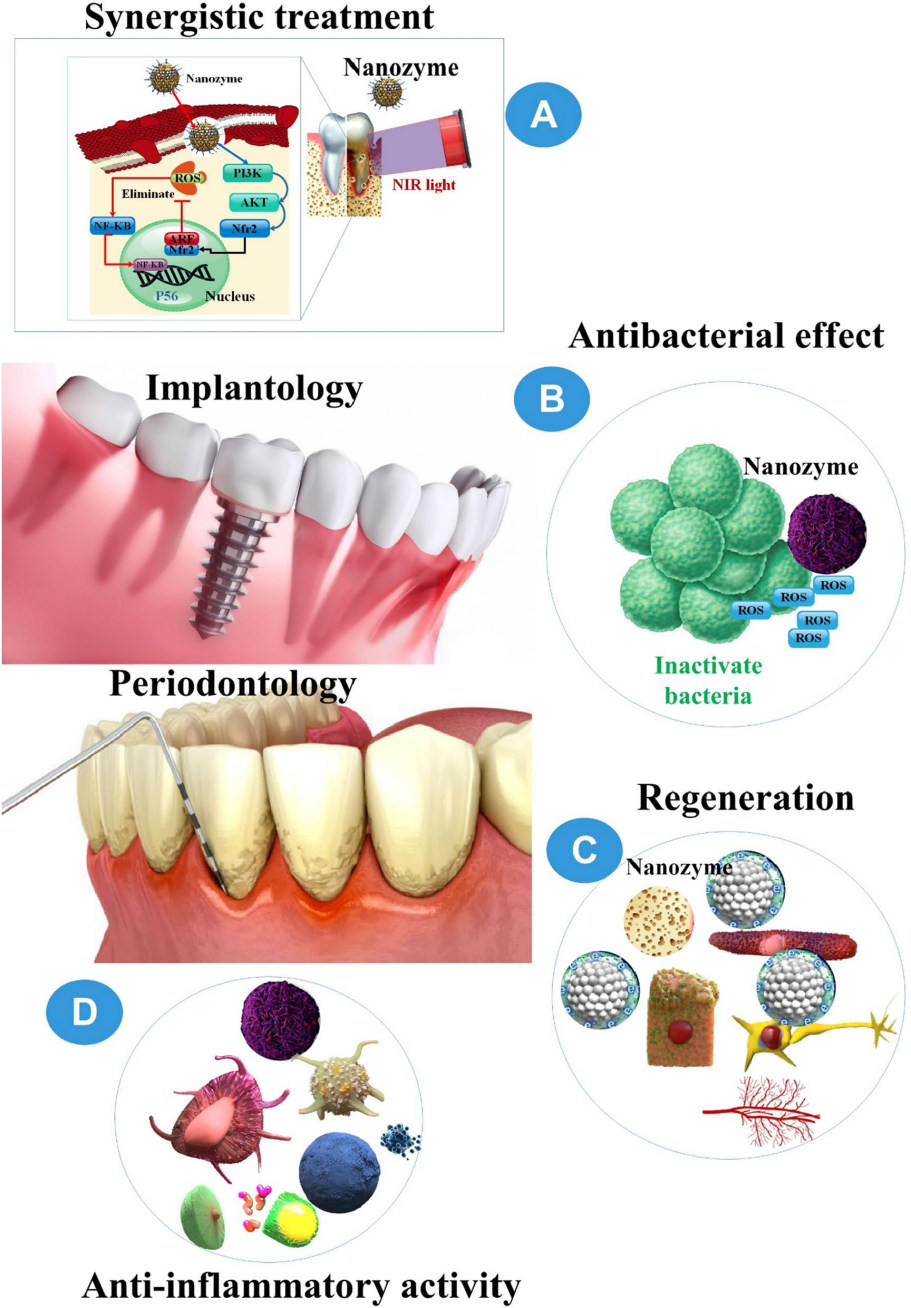
Effective applications in periodontology and implantology result from the **A** synergistic effects, **B** antibacterial, **C** regeneration-promoting, and **D** anti-inflammatory of nanozymes (NZs) that mimic enzyme functions. Several significant developments in the discipline are highlighted, in addition to forthcoming obstacles [41]

Despite the increased use of fluoride, the mainstay anticaries (protectants for tooth enamel), dental caries (tooth decay) remains the most prevalent human disease caused by oral biofilms, afflicting nearly half of the world's population, according to another study. In recent studies, it has been demonstrated that an iron oxide NZ formulation (Fer) that has been approved by the FDA can specifically and catalytically activate H_2_O_2_ to disrupt caries-causing biofilms; however, it does not exhibit the ability to interfere with enamel acid demineralization. The results of this investigation demonstrated that the combination of ferrous fluoride (Fe) and SnF_2_ inhibits biofilm accumulation and enamel degradation significantly more effectively than either element used alone. Unexpectedly, the data indicate that SnF_2_ substantially increases ROS production and antibiofilm activity while enhancing the catalytic activity of Fer. Fer, when combined with SnF_2_, demonstrates remarkable efficacy in the in vivo management of dental caries. It completely inhibits enamel demineralization and cavitation without inducing detrimental effects on host tissues or altering the diversity of the oral microbiota. Additionally, the combination of SnF_2_ and Fer increases its efficacy, resulting in comparable therapeutic effects at a fluoride concentration four times lower [106] (Fig. 4).

### Nanozyme in treatment of implant infections

Dental implants are the prevailing therapeutic modality utilized to address tooth loss and injury. Implant failure rates can reach 23% despite technological advances in treatment when peri-implantitis, a multispecies bacterial infection, is the underlying cause. As the number of implant placements increases by 8.78% annually, bacterial infection-related implant failure is a significant oral and general health concern. Implant failure is exacerbated by the increase in antibiotic resistance among oral microorganisms; therefore, adjunctive therapy is required to enhance implant outcomes [107]. Deep soft tissue infections associated with implants are notoriously challenging to treat with antibiotics due to their profound nature and frequent association with methicillin-resistant *S.aureus* (MRSA). Incision, drainage, and long-term administration of high-dose antibiotics are typically required to achieve this objective. However, it is more probable that these courses of action will facilitate the evolution of bacteria into superstrains [108, 109]. Biofilms, also referred to as bacterial communities, are considerably more difficult to eradicate from the surfaces of subsurface sites, including implants, due to their intrinsically high reproduction and strong adhesion [110, 111]. Antibiotic treatment failure is on the rise, and persistent systemic infections in human hosts are an unavoidable consequence. Thus, it is critical to devise in situ, antibiotic-free approaches that are efficacious in combating infections affecting deep tissues [112].

On the contrary, a novel era of potent tools to combat bacterial infections without inducing AMR has arrived with NZ-based antibacterial therapy. The antibacterial mechanism relies on enzyme-mimetic catalysis to produce exceedingly toxic agents, including ROS. The agents mentioned above can inflict rapid and irreversible harm upon the cell wall, membrane, DNA, and proteins of bacteria, in addition to extracellular DNA and biofilm polysaccharides [85, 113]. However, due to restricted substrate diffusion, the catalytic ROS production of NZs undoubtedly decreases in deep tissues. Deep infections necessitate NZs with increased activity to generate inhibitory levels of ROS at lower concentrations of substrate [68]. Following the efficacy of NZ treatment for periodontitis, there is considerable potential for the application of NZs in treating peri-implant diseases via modulation of the implant surface to enhance its antibacterial, anti-inflammatory, mechanical, and osteogenesis characteristics [41].

According to different research, the exceptional mechanical qualities and biocompatibility of Titanium (Ti) and its alloys have made them popular for usage as subcutaneous and percutaneous implants. Nevertheless, the accumulation of ROS and enduring inflammatory reactions at the implant location negatively impact the soft tissue integration of titanium implants, leading to several biological issues. This work used solvothermal and anodic oxidation to create Fe-nitrogen-doped carbon SAzymes (Fe-NC NZs) loaded Ti oxide nanotube arrays (Fe-NC@TNT) on medicinal Ti surfaces to solve this problem. Fe-NC@TNT was examined for its physical composition, surface morphology, enzyme-like catalytic activity, inflammatory response, and compatibility with soft tissues. By utilizing a distinctive nanotube array, the active sites of Fe-NC NZs are entirely exposed, leading to a substantial improvement in their enzyme-like catalytic capabilities. This enhancement eliminates superoxide anion, H_2_O_2_, and more hazardous ·OH. Consequently, this reduction in intracellular ROS levels in macrophages and fibroblasts effectively hinders inflammatory responses of macrophages and stimulates the functional expression of fibroblasts. Fe-NC@TNT has also been shown in vivo animal trials to successfully control the immune response and facilitate the integration of the implant with the surrounding soft tissues. The present study included the preparation of Ti oxide nanotube arrays (TNT) by anodic oxidation, which were then loaded with Fe-NC NZs on their surface via the polymerization reaction between formamide and Fe^3+^. Fe-NC NZs provided the samples with a significant antioxidant capacity and may further increase the hydrophilicity and corrosion resistance of TNT. They also efficiently scavenged superoxide anions, H_2_O_2_, and ·OH in the surrounding environment [114].

In addition to impairing the functions of osteogenic-relative cells, bacterial infection and the subsequent inflammatory response result in the ineffectiveness of Ti-based implantation. To treat this tissue, it is critical to develop multifunctional Ti implants (antimicrobial, anti-inflammatory, and pre-osteogenesis). In this study, TNTs were coated with zeolitic imidazolate frameworks-67 (ZIF-67) laden with osteogenic growth peptide (OGP) to produce a TNT-ZIF-67@OGP surface. The pH-sensitive ZIF-67@OGP coating underwent rapid dissolution in an acidic environment. Furthermore, the TNT-ZIF-67@OGP demonstrated potent antibacterial efficacy against *S. aureus*, MRSA, *E. coli*, and *S. mutans* due to ZIF-67 NP hydrolysis and the creation of an alkaline microenvironment. The implants exhibited excellent biocompatibility with macrophages and mesenchymal stromal cells (MSCs). Notably, in an inflammatory setting, TNT-ZIF-67@OGP may promote MSC cellular differentiation and reduce the inflammatory response. The in vivo research also showed that TNT-ZIF-67@OGP implants had potent antibacterial and anti-inflammatory characteristics early in the implantation process, which improved the late-stage osteointegration of the implant. Therefore, this multifunctional titanium implant that combines antibacterial and osteoimmunomodulatory properties is a good option for implant-associated infection bone regeneration [115].

According to different research, after implant-related surgery, the risk of biofilm-associated infections (BAIs) recurring is still high. It has been shown that biofilms on the surface of implants shield bacteria from antibiotics and thwart innate immune responses. Furthermore, there is still a lack of knowledge on removing lingering bacteria that might cause biofilm reinfection. This work reports a new "interference-regulation strategy" for fighting BAIs that uses bovine serum albumin-iridium oxide NPs (BIONPs) as an immunomodulator and biofilm homeostasis interrupter via ^1^O_2_-sensitized mild hyperthermia. By efficiently converting the abundant H_2_O_2_ in the biofilm microenvironment (BME) to sufficient O_2_, the CAT-like BIONPs can increase the production ^1^O_2_ when exposed to near-infrared irradiation. The perturbation of biofilm homeostasis induced by ^1^O_2_ (e.g., sigB, groEL, agr-A, icaD, eDNA) has the potential to disrupt the intricate defense mechanisms of biofilms, thereby increasing their susceptibility to mild hyperthermia. Furthermore, the bacterial membrane disintegration induced by moderate hyperthermia leads to protein leakage and ^1^O_2_ penetration, effectively eliminating bacteria within the biofilm. Following this, the immunosuppressive microenvironment re-rousing induced by BIONPs effectively re-orients macrophages to adopt a pro-inflammatory M1 phenotype in vivo, to consume any remaining biofilm, and to impede biofilm reconstruction. By combining ^1^O_2_-sensitized mild hyperthermia, immunotherapy, biofilm homeostasis interference, and mild hyperthermia, this approach offers a novel and efficacious method for treating refractory BAIs [116].

Because of compromised immune responses and antibiotic tolerance from bacterial biofilms, implant infections are challenging to treat with conventional antibiotic treatment. Therapeutic medicines must eradicate bacteria and control immune cell inflammation throughout the biofilm removal phase to effectively treat implant infections. Here, pH-responsive enzyme-like activities were used to construct multifunctional smart hollow Cu_2_MoS_4_ nanospheres (H-CMS NSs) that can self-adapt to eliminate biofilms and control macrophage inflammation in implant infections. The tissue milieu around implants becomes acidic during biofilm infection. Catalyzing the production of ROS that destroy bacteria directly and polarize macrophages toward a proinflammatory phenotype are H-CMS NSs with OXD/POD-like activities. Subsequently, ultrasound (US) irradiation can augment the POD-like activity and antibacterial characteristics of H-CMS NSs. Once biofilms have been eliminated, the tissue microenvironment surrounding implants becomes neutral rather than acidic. H-CMS NSs eradicate excessive ROS and exhibit CAT-like activity, thereby polarizing macrophages toward an anti-inflammatory phenotype and promoting infected tissue healing. This study presents a self-adaptive NZ that controls the immune response and antibiofilm activity by modulating the generation and elimination of ROS in response to the various pathological microenvironments encountered in implant infections throughout the therapeutic process [117].

In this study, researchers described Cu-doped CDs that exhibit increased catalytic (CAT-like, POD-like) activity in the oral environment. These CDs inhibit the initial bacterial adhesion of *S. mutans* and subsequently eradicate biofilms without causing harm to the surrounding oral tissues through the generation of ROS or O_2_. In particular, Cu-CDs have a strong affinity for peptidoglycans (PGN) and lipopolysaccharides (LPS). This gives them excellent antibacterial properties against Gram-positive (*S. aureus*) and Gram-negative (*E. coli*) bacteria, preventing wound purulent infection and accelerating wound healing. In addition, the Cu-CDs/H_2_O_2_ system exhibits superior tooth whitening performance compared to other alternatives, such as clinically utilized H_2_O_2_ and CDs, due to its negligible enamel and dentin degradation. The biocompatible Cu-CDs described in this study are expected to function as a potentially effective nano-mouthwash to remove oral pathogenic biofilms, promote wound healing, and whiten teeth. These results underscore the importance of Cu-CDs in the management of oral health [118] (Table 2).

**Table 2:** Potential utilizing of nanozymes in dental implant

Nanozymes	Antibacterial mechanism	Reference
CuN4-CNS	The findings obtained from both in vitro and in vivo experiments indicate that the ideal CuN4-CNS has great therapeutic effectiveness in treating both deep implant-related biofilm infections and superficial skin wounds by successfully inhibiting multidrug-resistant bacteria and eliminating recalcitrant biofilms	[68]
Fe-NC@TNT	Fe-NC@TNT was examined for its physical composition, surface morphology, enzyme-like catalytic activity, inflammatory response, and compatibility with soft tissues.Fe-NC@TNT has also been shown in vivo animal trials to successfully control the immune response and facilitate the integration of the implant with the surrounding soft tissues	[114]
TNT-ZIF-67@OGP	The TNT-ZIF-67@OGP demonstrated potent antibacterial efficacy against *S. aureus*, MRSA, *E. coli*, and *S. mutans* due to ZIF-67 NP hydrolysis and the creation of an alkaline microenvironment	[115]
BIONPs	Reports a new "interference-regulation strategy" for fighting BAIs that uses bovine serum albumin-iridium oxide NPs (BIONPs) as an immunomodulator and biofilm homeostasis interrupter via singlet oxygen (^1^O_2_)-sensitized mild hyperthermia	[116]
Cu_2_MoS_4_	pH-responsive enzyme-like activities were used to construct multifunctional smart hollow Cu_2_MoS_4_ nanospheres (H-CMS NSs) that can self-adapt to eliminate biofilms and control macrophage inflammation in implant infections	[117]
Cu-CDs/H_2_O_2_	Antibacterial properties against both Gram-positive (*S. aureus*) and Gram-negative (*E. coli*) bacteria, preventing wound purulent infection and accelerating wound healing. In addition, the Cu-CDs/H_2_O_2_ system exhibits superior tooth whitening performance compared to other alternatives, such as clinically utilized H_2_O_2_ and CDs, due to its negligible enamel and dentin degradation	[118]

### Nanozyme in treatment of peri-implantitis

A site-specific infectious condition called peri-implantitis results in soft tissue inflammation and bone loss surrounding an osseointegrated implant when it is not functioning correctly. The state of the surrounding tissue, the implant's design, its degree of roughness, its external morphology, and an excessive mechanical strain all influence the etiology of implant infections. Spirochetes and mobile Gram-negative anaerobes are the bacteria most often linked to implant failure unless the cause is a straightforward mechanical overload [119]. When an osseointegrated implant is not operating as intended, the surrounding soft tissue becomes inflamed, and bone is lost, a condition known as peri-implantitis. The two categories of current techniques should be surface NZ modification of implants or local NZ therapy. Numerous researchers have focused on altering the surface of implants to enhance clinical results. In particular, they have prepared various physical and chemical alterations to enhance the osseointegration between the implant surface and alveolar bone. Additionally, improving osseointegration is a goal of the bioactive coatings that encourage the adhesion and colonization of cells and proteins relevant to osseointegration. Meanwhile, enhancing the implant surface's antibacterial capabilities may prevent bacterial adherence and activity, preventing implant-related inflammation [120]. To combat the bacterial pathogen epidemic, alternative antibacterial medicines based on ultrasound (US) have recently been investigated. A great alternative is antimicrobial sonodynamic treatment (aSDT), which uses US irradiation to create ROS and accomplish antibiotic-free mediated antimicrobial actions. Furthermore, aSDT has tremendous promise in treating deep infections because of its greater tissue penetrability of US compared to light irradiation. While ROS generation for antimicrobial activity is achievable with conventional sonosensitizers, unsatisfactory sterilization in aSDT is caused by several constraints, including limited penetration rate, nonspecific dispersion, and poor ROS production under hypoxic settings. As high-performance agents in aSDT, newly developed nanosonosensitizers provide significant benefits over traditional sonosensitizers, as previously mentioned. Thus, controlling bacterial infections by nanosonosensitizer-mediated aSDT has a promising future [121]. Host immune systems, which function as critical barriers against biofilm-associated implant infections, are vital resistance mechanisms. However, biofilms impede the entry of antibacterial species, obstruct the phagocytosis of immune cells, and thwart inflammatory responses of the host, ultimately undermining the ability of the host immune system to eliminate biofilms. Through the encapsulation of erythrocyte membrane fragments on the surface of microbubbles fabricated from Fe_3_O_4_ NPs and subsequent loading with hydroxyurea (EMB-Hu), a cell-like construct is created. When stimulated with the US, EMB-Hu endures a stable oscillation process that functions as an “exocytosis” mechanism. This mechanism facilitates the disruption of biofilm, the release of agents, and the enhancement of the penetration of catalytically generated anti-bacterial species within biofilms. Furthermore, EMB-Hu-stimulated “exocytosis” induced by the US can enhance macrophage phagocytosis and pro-inflammatory macrophage polarization, both of which are essential for the removal of disrupted biofilms. In summary, this research has demonstrated the utilization of cell-like microbubbles containing “exocytosis” mechanisms stimulated by the US to traverse the biofilm barrier and activate macrophages in an inflammatory response to methicillin-resistant *Staphylococcus aureus* (MRSA) biofilms-induced implant infections. As a result, beneficial therapeutic outcomes have been achieved [122]. Environmentally sensitive therapeutic platforms with low dose-limiting toxicity, good selectivity, and low drug resistance have attracted much attention. When antibacterial activity in therapeutics is activated on demand by exogenous or endogenous triggers, they may demonstrate remarkable therapeutic results. Ultrasound, microwaves, light, and magnetism are examples of external stimuli. Most endogenous stimuli are pathological characteristics of bacterial infections, such as acidic pH, altered enzymatic activity, and aberrant temperature [123].

Despite the exponential annual growth in dental implant procedures, peri-implantitis continues to be a significant concern for numerous physicians. Under the influence of bacteria, peri-implantitis is an inflammatory reaction of the tissue surrounding the implant; it is the leading cause of dental implant failure. To mitigate the risk of peri-implantitis, it is crucial that patients practice appropriate oral hygiene and that their dentists select suitable implant materials and designs (e.g., only implant when the patient's periodontal health is stable). NZs, on the other hand, are can rapidly protect the tissue surrounding the implant from peri-implantitis by their antibacterial properties. Moreover, numerous light-responsive NZs can treat peri-implantitis when exposed to light. Thukkaram et al. discovered that Fer coating inhibits bacterial vitality and prevents bacterial adhesion to the surface of biological materials [124, 125]. Produced using chemical microwave technology, the controllable ultrafine CeO_2_ NPs are capable of penetrating cells and producing O_2_ free radicals, which inhibit the development of microorganisms. Interleukins and inflammatory factors contribute to peri-implantitis. In contrast to Ce^4+^, Ce^3+^ exhibits enhanced SOD activity and a more robust capacity for ROS removal. Li et al. synthesized an unprecedented octahedral CeO_2_ with an elevated Ce^3+^ value. The application of octahedral CeO_2_ coating onto the implant's surface effectively impedes the initial colonization of *S. sanguis*, thereby suppressing the development of plaque biofilm. Applying NZs onto implants has demonstrated remarkable antibacterial and anti-inflammatory properties, suggesting that they could be utilized to eradicate peri-implantitis [32]. Another research revealed that one of the main reasons dental implants fail is peri-implantitis. An extensive decline in oral health results from bacterial biofilm contamination on the implant, which causes soft tissue irritation and adjacent bone resorption. On decontaminated implant surfaces, however, re-osseointegration cannot be induced by standard biofilm removal techniques such as mechanical cleaning and antiseptic treatment. This results from two factors: (1) decontamination procedures that fail to altogether remove biofilm from inaccessible areas and (2) modifications to the physicochemical properties of implant surfaces. Researchers presented a novel therapeutic strategy for peri-implantitis that is both safe and efficacious. The method entails decontaminating biofilms adhered to implants by utilizing the kinetic energy of microsized O_2_ bubbles produced by a catalytic reaction involving manganese oxide (MnO_2_) NZ sheet-doped silica diatom microparticles (Diatom Microbubbler, DM). Compared to conventional antiseptics like chlorhexidine or 3% H_2_O_2_ when used alone, rapidly moving microsized DM particles can penetrate narrow spaces between implant screws, exerting just the right amount of force to destroy biofilms without harming the surrounding mucosa or implant surfaces. As a result, DM cleaning on the implant surface impacted by peri-implantitis promotes effective re-osseointegration. In conclusion, our novel DM-based treatment strategy will emerge as a viable substitute to address clinically complex peri-implantitis issues [126].

The investigators of this study present a new approach to biofilm removal that does not involve antibiotics. They suggest a BME-responsive double-layered MOF bionanocatalysts (MACG) made of MIL-100 and CuBTC as a synergistic bionanocatalysts-driven heat-amplified chemodynamic therapy (CDT) and innate immunomodulation. It is possible to release GOx and an activable photothermal agent, 2,2'-azino-bis (3-ethylbenzothiazoline-6-sulfonic acid) (ABTS), sequentially once CuBTC has degraded due to acidity at the acidic BME. GOx breaks down glucose into gluconic acid and H_2_O_2_, which might further acidify the BME and hasten the release of GOx and ABTS, as well as the breakdown of CuBTC. The findings, both in vitro and in vivo, demonstrate that MIL-100, which mimics horseradish POD (HRP), may catalyze the oxidation of ABTS into oxABTS when self-supplied H_2_O_2_ is present. This produces a photothermal impact that damages eDNA and disrupts the biofilm structure. In addition to depleting glutathione, the Cu ion released from the broken down CuBTC may also split H_2_O_2_ into OH, which can efficiently penetrate heat-induced loose biofilms and kill sessile bacteria (up to 98.64%), including MRSA and *E. coli*. Specifically, by secreting pro-inflammatory cytokines (e.g., IL-6, TNF-α, etc.) and creating a persistently pro-inflammatory milieu in peri-implant biofilm-infected rats for at least 14 days, MACG-stimulated M1-macrophage polarization reduces the biofilm regeneration. With minimal side effects, this BME-responsive approach has the potential to eradicate resistant peri-implant biofilm infections accurately [127].

According to another study, peri-implant infection induced by bacterial biofilm constitutes the primary cause of failed dental implant repairs. The efficacy of infection control primarily hinges on the eradication of bacterial biofilm. However, as bacterial resistance increases, traditional medicine treatments become impractical. Ultrasound-activated antibacterial sonodynamic therapy (aSDT) has gained recognition in recent times as a promising approach to the treatment of biofilm infections. For aSDT, an activatable nanoplatform (Au-TNT) fabricated on the implant’s surface is proposed in this investigation. Under ultrasonic irradiation, Au-TNT could swiftly generate O_2_, thereby alleviating the hypoxic microenvironment of biofilm and enhancing the anti-biofilm efficacy of aSDT. In addition, it can produce ·OH and ^1^O_2_, which confer an exceptionally potent antibacterial effect against pathogenic biofilms of various species, as determined by bacterial survival rate, cell membrane rupture, biofilm metabolism, and thickness. In contrast, Au-TNT demonstrated remarkable antibacterial efficacy in vivo, as evidenced by a three-log reduction in colony-forming units (CFU) compared to the control group. Notably, the results also demonstrated that Au-TNT inhibited the expression of inflammatory factors and stimulated bone repair. Therefore, this research presents a nanoplatform catalyzed by sonodynamics that effectively eliminates biofilm and treats peri-implant infections. By loading Au NPs onto TNT, researchers have proposed a simple and long-lasting antibacterial system for dental implant surfaces. As a sonosensitizer, TNT produced by anodization on Ti implants was utilized; introducing Au NPs improved their catalytic performance. Electrons migrate from the TNT to the Au NPs in response to US irradiation, thereby preventing the recombination of electron–hole pairs and increasing the yields of O_2_ and ROS [128].

An alternative study proposes a safe and efficacious therapeutic strategy for peri-implantitis that utilizes the kinetic energy of microsized O_2_ bubbles produced by the catalytic reaction of manganese oxide (MnO_2_) NZ sheet-doped silica DM to decontaminate implant-bound biofilms. As opposed to conventional antiseptics like chlorhexidine or 3% H_2_O_2_ when used alone, rapidly moving microsized DM particles are capable of penetrating the narrow spaces between implant screws and exerting precisely the right amount of force to destroy biofilms without harming the surrounding mucosa or implant surfaces. Decontamination with DM promotes re-osseointegration on the surface of the implant afflicted by peri-implantitis [126].

Peri-implantitis can be exacerbated and peri-implant tissue regeneration can be hindered, because sustained pathological stimuli can accelerate macrophage-mediated inflammation, facilitated by the microgap between the implant and surrounding connective tissue. The abutment, being the transmucosal component of the implant, must be biofunctionalized to facilitate the restoration of the gingival barrier. An implant abutment coating inspired by mussel biology, which comprises tannic acid (TA), cerium, and minocycline (TA-Ce-Mino), is described in this article. To facilitate cell adherence, pyrogallol, and catechol groups are introduced by TA. In addition, the enzyme-mimetic activity of the Ce^3+^/Ce^4+^ conversion to remove ROS while producing O_2_ promotes the polarization of anti-inflammatory M2 macrophages, which aids in forming a regenerative environment. On the TA surface, minocycline is utilized to generates local drug storage for responsive antibiosis. Furthermore, the therapeutic mechanism underpinning the coating's exogenous and endogenous antioxidative effects is elucidated: exogenous antioxidation is facilitated by the inherent properties of Ce and TA; endogenous antioxidation is achieved by promoting antioxidants and maintaining mitochondrial homeostasis. Furthermore, it incites integrin activation, which enhances VEGF-mediated angiogenesis and tissue regeneration via the PI3K/Akt and RhoA/ROCK pathways. By integrating multidimensional orchestration and antibiosis, TA-Ce-Mino effectively restores function to effector cell differentiation and soft tissue barriers, thus creating an immune microenvironment impervious to pathogen invasion. This study therefore offers crucial insight into the biological mechanism and design of abutment surface modification for peri-implantitis prevention [129].

**Fig. 5:**
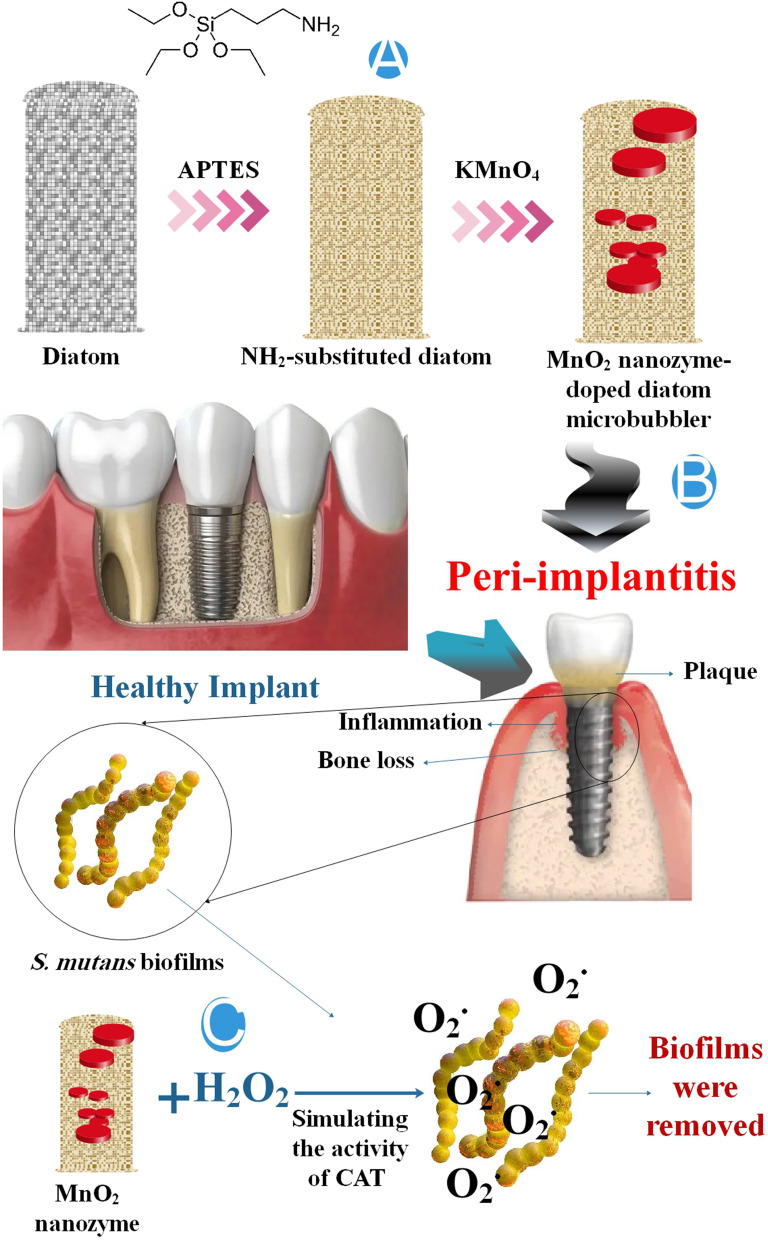
MnO_2_ nanozyme-doped diatom microbubbler (DM) antibacterial effects in peri-implantitis. **A** MnO_2_ NZ-doped DM was recently developed. **B** and **C** By simulating the activity of CAT in H_2_O_2_ solution, DM is capable of producing oxygen and moving with the ejection of oxygen microbubbles, thereby creating a mechanical self-cleaning effect. CHX eradicated *S. mutans* but was incapable of eliminating the majority of biofilms on brackets [130]

Using orthodontic brackets fosters the development of *S. mutans* biofilm, thereby augmenting the likelihood of developing dental caries and white spots. For the eradication of biofilm, a MnO_2_ NZ-doped DM was recently developed. By simulating the activity of CAT in an H_2_O_2_ solution, DM is capable of producing O_2_ and moving with the ejection of O_2_ microbubbles, thereby creating a mechanical self-cleaning effect. Following the protocol, DM was prepared and examined with a scanning electron microscope (SEM). *S. mutans* biofilms were subjected to various treatments, including phosphate-buffered saline (PBS) for the PBS group, 0.12% chlorhexidine for the CHX group, 3% H_2_O_2_ for the H_2_O_2_ group, and co-treatment with 3% H_2_O_2_ and 3 mg/mL of DM for the DM group. The results of the crystal violet assay indicated that the DM group eliminated biofilms more efficiently than the CHX group and that the CHX group eliminated a greater quantity of biofilms than the control group. According to SEM and CLSM images, CHX eradicated *S. mutans* but was incapable of eliminating the majority of biofilms on brackets. On debonded brackets, DM successfully eliminated biofilms and mature multispecies biofilms [130] (Fig. 5) (Table 3).

**Table 3:** Antibacterial effects of several nanozymes in peri-implantitis

Nanozymes	Antibacterial mechanism and function	Reference
Manganese Oxide Nanozyme-Doped Diatom	The method entails decontaminating biofilms adhered to implants by utilizing the kinetic energy of microsized oxygen bubbles produced by a catalytic reaction involving manganese oxide (MnO_2_) nanozyme sheet-doped silica diatom microparticles (Diatom Microbubbler, DM)	[126]
TA-Ce-Mino	A mussel-bioinspired implant abutment coating containing tannic acid (TA), cerium, and minocycline (TA-Ce-Mino) is reported. TA provides pyrogallol and catechol groups to promote cell adherence. Besides, Ce^3+^/Ce^4+^ conversion exhibits enzyme-mimetic activity to remove reactive oxygen species while generating O_2_	[129]
CDT	A novel non-antibiotic strategy based on the synergy of bionanocatalysts-driven heat-amplified chemodynamic therapy (CDT) and innate immunomodulation is proposed for specific biofilm elimination by the smart design of a biofilm microenvironment (BME)-responsive double-layered metal–organic framework (MOF) bionanocatalysts (MACG) composed of MIL-100 and CuBTC	[127]
Au-TNT	Ultrasound-activated antibacterial sonodynamic therapy (aSDT) has gained recognition in recent times as a promising approach to the treatment of biofilm infections. For aSDT, an activatable nanoplatform (Au-TNT) fabricated on the implant's surface is proposed in this investigation. Under ultrasonic irradiation, Au-TNT could swiftly generate O_2_, thereby alleviating the hypoxic microenvironment of biofilm and enhancing the anti-biofilm efficacy of aSDT	[128]
A MnO_2_ nanozyme-doped diatom microbubbler (DM)	For the eradication of biofilm, a MnO_2_ nanozyme-doped DM was recently developed. By simulating the activity of CAT in H_2_O_2_ solution, DM can produce oxygen and moving with the ejection of oxygen microbubbles, thereby producing a mechanical self-cleaning effect	[130]

### Nanozyme in treatment of periodontitis

Periodontitis is a chronic inflammatory disease induced by the invasion of periodontal tissues by bacteria in dental detritus. As the condition advances, it frequently induces periodontal pocket formation, loosening of teeth, and receding gums, all contributing to the eventual demise of the affected teeth. More than ten percent of the world's population suffers from severe periodontitis [131]. As antibiotics are generally ineffective against biofilms, the prevailing clinical approach to managing periodontitis involves a dual-pronged approach involving antibiotic therapy and mechanical debridement. This strategy aims to eliminate the bacteria in the periodontal pocket before eliminating the biofilm. In contrast, mechanical debridement frequently results in patient discomfort, hemorrhage, and gingival damage. In contrast, the antimicrobial effect of antibiotics is gradual and susceptible to loss in the oral environment, necessitating frequent administration. Furthermore, the proliferation of antibiotic usage may give rise to bacterial resistance, an even more severe dilemma. Therefore, it is necessary to develop an antibiotic-free, non-invasive, rapid, and effective anti-biofilm treatment [132]. Periodontitis is an inflammatory condition distinguished by the resorption of alveolar bone and tooth loss. Periodontitis is initially caused by bacteria, and an excess of ROS promotes and exacerbates inflammation [133]. The escalating global incidence of periodontal and peri-implant diseases has garnered considerable interest. NZs, which possess enzyme-like activity and are multifunctional nanomaterials, have established a presence within the biomedical domain. NZs have significantly advanced research in the fields of periodontics and implantology, specifically about the maintenance of periodontal health and the enhancement of implant success rates. Review NZs for antimicrobial therapy, anti-inflammatory therapy, promotion of tissue regeneration, and synergistic effects in periodontal and peri-implant diseases to underscore this development [41].

**Fig. 6:**
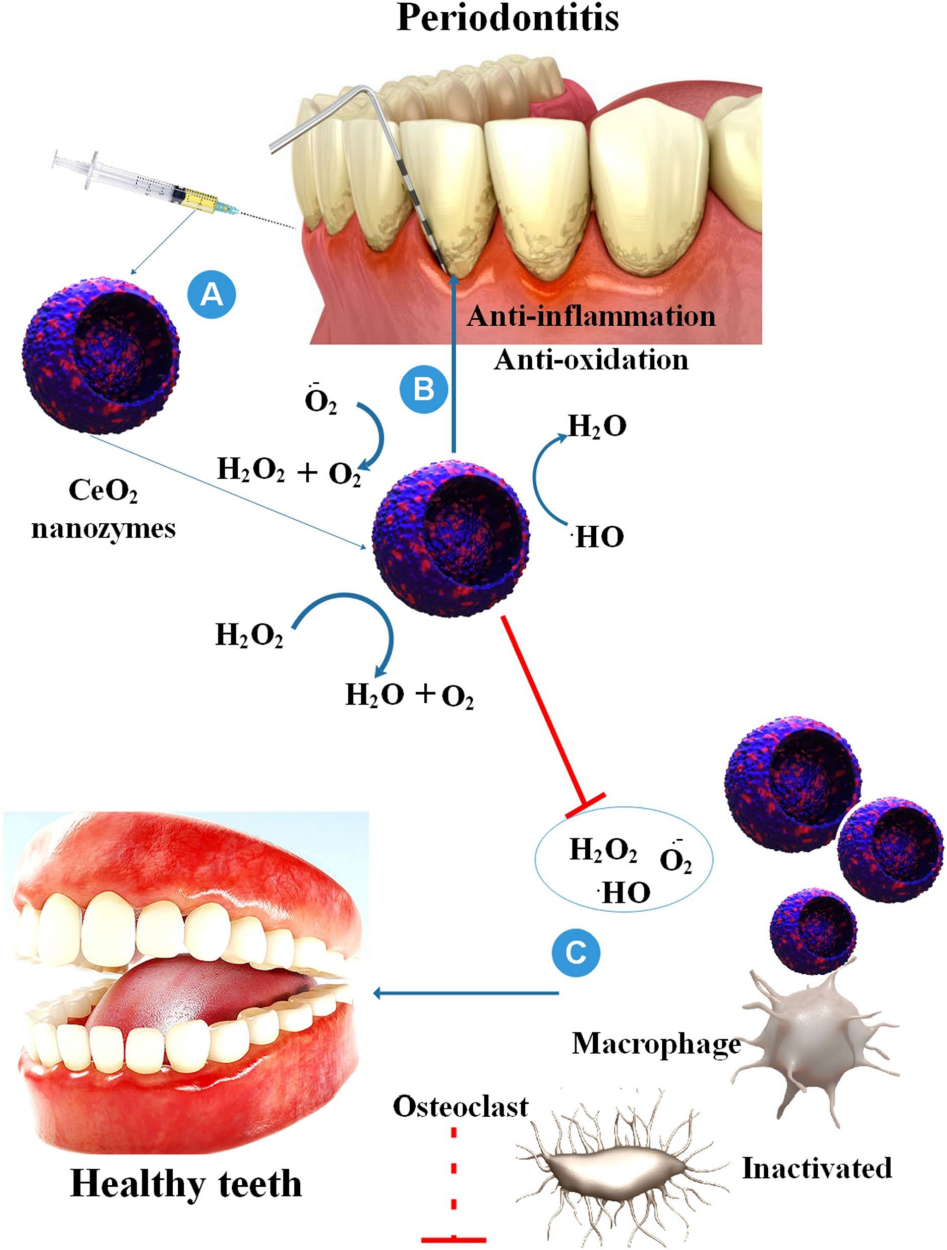
A schematic representation of the therapeutic impact of CeO_2_ nanoenzymes on periodontitis [65]

Researchers presented an in-situ injection of CeO_2_ NPs as a therapeutic approach for managing periodontitis in this study. Furthermore, ideal results could be achieved by synthesized CeO_2_ NPs functioning as ROS scavengers in the inflammatory microenvironment. Experiments in vivo and in vitro provide substantial evidence that CeO_2_ NPs scavenge multiple ROS and inhibit lipopolysaccharide-stimulated ROS-induced inflammatory responses. Additionally, CeO_2_ NPs can impede the MAPK–NFκB signaling pathway, thereby inhibiting inflammatory factors. Furthermore, the findings obtained from a rodent model of periodontitis indicate that CeO_2_ NPs can significantly inhibit alveolar bone resorption, reduce osteoclast activity and inflammation, and thus enhance the regeneration of damaged tissues. In its entirety, the current investigation highlights the promising prospects of CeO_2_ NPs as a therapeutic agent for periodontitis and offers significant knowledge regarding the utilization of NZs in inflammatory disorders. As a result, CeO_2_ NPs with high CAT-like and SOD-like activity, in addition to ˙OH scavenging capability, were synthesized. ROS scavenging activity was demonstrated by these NPs in vitro and in vivo. Furthermore, their anti-inflammatory and antioxidant properties were shown by their ability to inhibit the MAPK–NFκB signaling pathway and activate the Nrf2–HO-1 pathway, respectively. In a rat periodontitis model, CeO_2_ NPs were found to inhibit inflammation and bone loss. Consequently, CeO_2_ NPs exhibit considerable potential in clinically treating periodontitis [65] (Fig. 6).

**Fig. 7:**
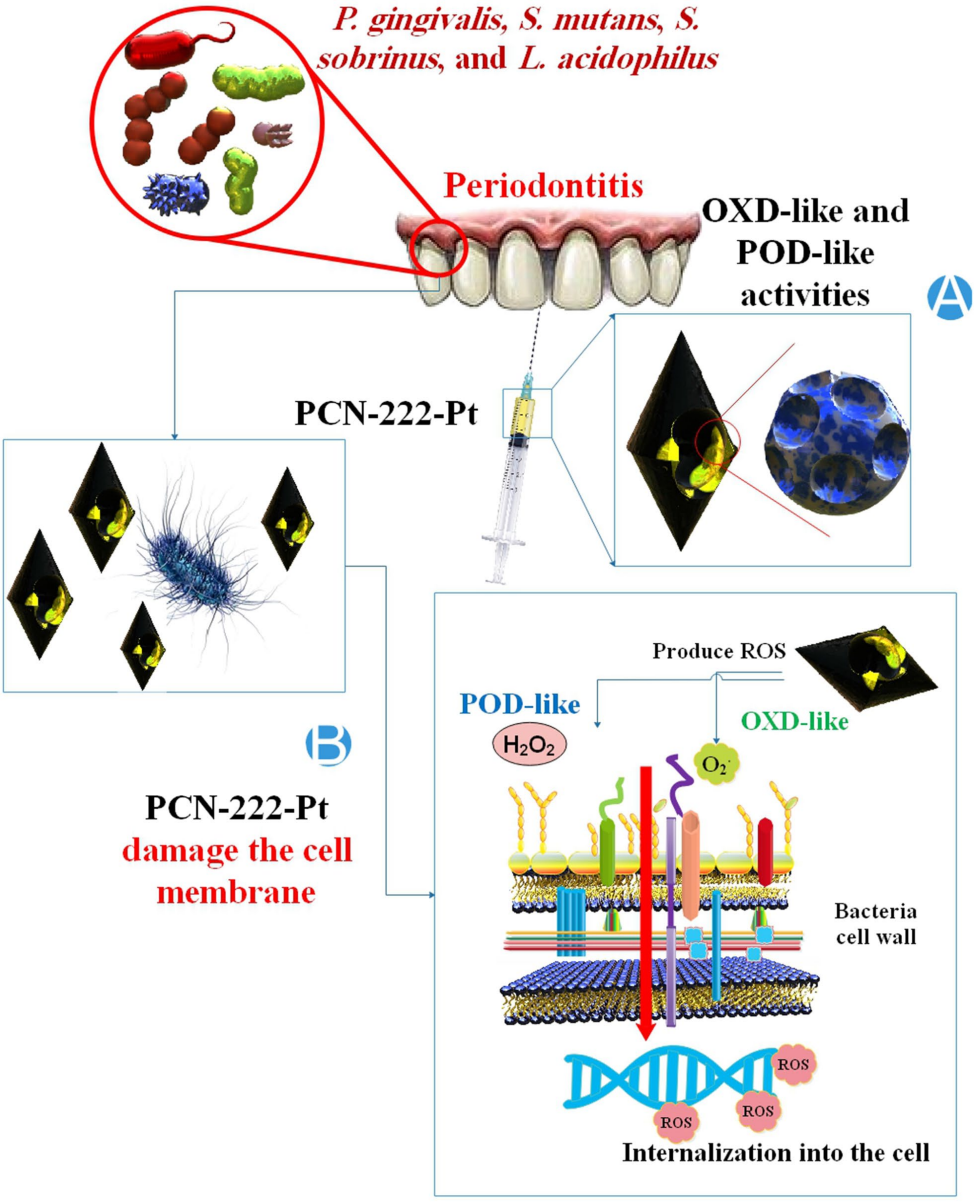
Theoretical evaluation of MOF-based single-atom catalysts for periodontitis treatment. **A** Schematic of MOF-based single-atom catalysis systems and a porphyrin metal–organic framework (PCN-222-Pt). **B** PCN-222-Pt, which generates ROS spontaneously and exhibits potent OXD-like and POD-like activities, demonstrates exceptional anti-biofilm efficacy (98.69% against *S. aureus* biofilm, 99.91% against *E. coli* biofilm) in vitro within one hour [134]

An additional study demonstrated that through ongoing investigation, catalysis systems based on MOFs and single-atom catalysis have emerged, offering more extensive potential for implementation in biological contexts. MOF-based catalysis systems have more catalytic sites than conventional catalysis systems due to their 3D and highly porous structure. By employing metal atom dispersion, single-atom catalysis systems achieve significantly greater catalytic activity while consuming significantly less metal. Given this consideration, researchers postulated that introducing single atoms possessing enzyme-like activity into MOF could substantially enhance its catalytic activity. Single-atom dopped MOF-based catalysis systems for the treatment of biofilm-induced periodontitis are described in this article. In this study, the researchers aim to develop an injectable ointment that exhibits potent anti-biofilm activity and favorable biocompatibility. To achieve this, they will utilize MOF-based single-atom catalysis systems and a porphyrin metal–organic framework (PCN-222-Pt) infused with Pt single atoms. Using theoretical screening, it has been determined that incorporating single metal atoms (Pt, Au, Cu, Ru) into PCN-222 can enhance its OXD-like activity, thereby diminishing the adsorption and activation energies of O_2_. PCN-222-Pt, which generates ROS spontaneously and exhibits potent OXD-like and POD-like activities, demonstrates exceptional anti-biofilm efficacy (98.69% against *S. aureus* biofilm, 99.91% against *E. coli* biofilm) in vitro within one hour. In contrast to the clinically prescribed periocline, the injectable PCN-222-Pt ointment demonstrated a reduced bone degradation rate, healthier periodontal tissue, and an alleviation of inflammation response in treating biofilm-induced periodontitis. Without antibiotics, this work presents a rapid, effective, non-invasive, and practical method for treating periodontitis [134] (Fig. 7).

An additional investigation demonstrated that oral diseases induced by pathogenic biofilms, including *F. nucleatum*-induced periodontitis, have a substantial effect on human health. Despite the implementation of scaling and antibiotics, clinical treatment for diseases caused by oral biofilm continues to face obstacles such as unavoidable hemorrhage, drug resistance, and inadequate therapeutic efficacy. In recent times, the emergence of NZ has presented a novel approach to the inhibition and elimination of oral biofilms, and by replacing Pt atoms in the structure of Au/Pt NCs researchers created a bimetallic clusterzyme with enhanced POD-like activity. The enzyme exhibited a high catalytic activity towards H_2_O_2_ due to the synergistic effect between Au and Pt atoms. With the coupling of GOX to Au/Pt NCs (Au/Pt NCs@GOX), a clusterzyme was developed that exhibited excellent biocompatibility and self-promoting antibacterial properties. This clusterzyme took advantage of the nutrient-rich oral environment to catalytically convert nontoxic glucose into highly toxic ·OH via a cascade reaction. Consequently, it effectively inhibited and eliminated biofilm induced by *F. nucleatum* in vivo. Additional evidence was obtained from in vivo animal experiments demonstrating that the Au/Pt NCs@GOX clusterzyme effectively and safely treated periodontitis in rats, inhibited inflammation, and stimulated periodontal tissue regeneration. Overall, this study's cascade clusterzyme offers a promising avenue for the future clinical application of a safe and effective method to treat oral biofilm-induced periodontitis. In addition, this cascade catalytic system was developed to eliminate biofilm induced by *F. nucleatu*m and eliminate planktonic *F. nucleatum*. In conclusion, the therapeutic efficacy of the Au/Pt NCs@GOX catalytic system against *F. nucleatum*-induced periodontitis was assessed using a periodontitis model. Biocompatibility and exceptional antibacterial and antibiofilm activity confer a promising application outlook for the ultra-small clusterzyme in treating oral diseases [38].

Periodontitis, according to another study, is a chronic inflammatory disease caused by dental plaque that destroys periodontal tissues due to the excessive accumulation of ROS, matrix metalloproteinase (MMP), and other substances. Currently, the primary therapeutic approaches—including local mechanical debridement and antibiotic delivery—face challenges in effectively addressing the persistent bacterial biofilm, mitigating the excessive inflammatory response, and regenerating the damaged periodontal tissues. The TM/BHT/CuTA hydrogel system, which is composed of Cu-based NZs (Cu tannic acid coordination nanosheets, CuTA NSs) and triglycerol monostearate/2,6-di-tert-butyl-4-methylphenol (TM/BHT) hydrogel, has been proposed by researchers. By retaining the inflammatory sites with a positive charge via electrostatic adsorption, the negatively charged TM/BHT/CuTA can hydrolyze in response to the increasing MMP of periodontitis, thereby enabling the on-demand release of the CuTA NZ. CuTA NZ, which was liberated, possesses antibacterial and antiplaque properties. In contrast, it can scavenge numerous ROS by simulating the cascade process of SOD and CAT as a metal-phenolic NZ. Moreover, by modulating macrophage polarization from M1 to M2 via the Nrf2/NF-κB pathway, the CuTA NZ alleviates inflammation and expedites tissue regeneration in the context of periodontitis by decreasing pro-inflammatory cytokines, increasing anti-inflammatory cytokines and promoting the expression of osteogenetic genes in that order. In its entirety, the TM/BHT/CuTA multifunctional NZ on-demand release platform presents an advantageous approach to managing periodontitis [135].

The gradual integration of oxidoreductase NZs for ROS regulation into periodontology treatments has been documented in another study. Existing NZs for the treatment of periodontitis, on the other hand, eliminate ROS in a broad and non-specific manner, disregarding their physiological functions as they occur naturally. This may lead to uncontrolled adverse effects. By employing the MIL-47(V)-F (MVF) NZs, which emulates the activity of glutathione POD (GPx), this research suggests that ROS can be effectively regulated through the targeted elimination of H_2_O_2_, the most abundant ROS. MVF promotes periodontal regeneration, controls inflammation, and regulates the immune microenvironment using H_2_O_2_ elimination. Additionally, MVF directly promotes the osteogenic differentiation of periodontal stem cells, which is facilitated by the vanadium content of MVF. By activating the Nrf2/HO-1 pathway, MVF regulates ROS. Additionally, it directly stimulates osteogenic differentiation via the phosphatidylinositol 3-kinase/protein kinase B (PI3K/Akt) pathway. GPx-mimicking NZs are utilized to establish a prospective periodontitis therapy strategy by their threefold effects—antioxidation, immunomodulation, and regulation of bone remodeling—which make NZs an ideal instrument for advancing precision medicine [136].

A separate investigation devised a novel FeSN, which was produced by doping histidine-doped FeS_2_ and exhibited significant POD-like activity to eliminate oral biofilm and manage periodontitis. FeSN demonstrated a remarkably high level of POD-like activity, with theoretical calculations and enzymatic reaction kinetics indicating that its catalytic efficiency was around thirty times greater than that of FeS_2_. In the presence of H_2_O_2_, the antibacterial experiments demonstrated that FeSN exhibited potent antibacterial activity against *F. nucleatum*. This was evidenced by increased OXD coenzyme levels and a decrease in glutathione reductase and ATP levels in bacterial cells. FeSN's exceptionally high POD-like activity facilitated the identification of pathogenic biofilms and stimulated the degradation of biofilm structure. In addition, FeSN exhibited remarkably low cytotoxicity and remarkable biocompatibility with human fibroblast cells. FeSN exhibited substantial therapeutic effects in a rodent periodontitis model through the reduction of biofilm formation, inflammation, and alveolar bone loss. Collectively, researchers’ findings indicated that FeSN, which was produced via the self-assembly of two amino acids, offered considerable potential as a strategy for the elimination of biofilm and the treatment of periodontitis. This approach can surmount the constraints of existing therapeutic modalities and furnish a viable substitute for the management of periodontitis [30].

To improve the activity resembling POD, researchers created a bimetallic clusterzyme in this study by substituting Pt atoms in the structure of Au/Pt NCs. As a result of the synergistic effect between Au and Pt atoms, this enzyme exhibited a high catalytic activity towards H_2_O_2_. By coupling Au/Pt NCs@GOX, a clusterzyme with excellent biocompatibility and self-promoting antibacterial effect was developed in response to the lack of catalytic activity in near-neutral conditions and the need for a high H_2_O_2_ concentration. This clusterzyme could fully exploit the nutrient-rich oral environment to convert nontoxic glucose into highly toxic ·OH via a cascade catalytic reaction, thus impeding and eliminating biofilm induced by F. nucleatum in viability. In addition, animal experiments in vivo demonstrated that the Au/Pt NCs@GOX clusterzyme could effectively treat periodontitis in rodents, reduce inflammation, and stimulate the regeneration of periodontal tissue while remaining safe to use. In conclusion, the cascade clusterzyme described in this study offers a method for future clinical implementation that is both safe and effective in treating oral biofilm-induced periodontitis [38] (Table 4).

**Table 4:** Antibacterial effect of different nanozymes in periodontitis

Nanozymes	Function	Reference
CeO_2_	Experiments in vivo and in vitro provide substantial evidence that CeO_2_ NPs scavenge multiple ROS and inhibit lipopolysaccharide-stimulated ROS-induced inflammatory responses. As a result, CeO_2_ NPs with high CAT-like and SOD-like activity, in addition to ˙OH scavenging capability, were synthesized	[65]
PCN-222-Pt	PCN-222-Pt, which generates ROS spontaneously and exhibits potent OXD-like and POD-like activities, demonstrates exceptional anti-biofilm efficacy (98.69% against *S. aureus* biofilm, 99.91% against *E. coli* biofilm) in vitro within one hour. In contrast to the clinically prescribed periocline, the injectable PCN-222-Pt ointment demonstrated a reduced bone degradation rate, healthier periodontal tissue, and an alleviation of inflammation response in treating biofilm-induced periodontitis	[134]
Au/Pt NCs@GOX	Evidence was obtained from in vivo animal experiments demonstrating that the Au/Pt NCs@GOX clusterzyme effectively and safely treated periodontitis in rats, inhibited inflammation, and stimulated periodontal tissue regeneration	[38]
TM/BHT/CuTA	The TM/BHT/CuTA hydrogel system, which is composed of Cu-based nanozymes (Cu tannic acid coordination nanosheets, CuTA NSs) and triglycerol monostearate/2,6-di-tert-butyl-4-methylphenol (TM/BHT) hydrogel, has been proposed by researchers	[135]
MIL-47(V)-F (MVF)	By employing the MIL-47(V)-F (MVF) nanozyme, which emulates the activity of glutathione POD (GPx), this research suggests that ROS can be effectively regulated through the targeted elimination of H_2_O_2_, the most abundant ROS. MVF promotes periodontal regeneration, controls inflammation, and regulates the immune microenvironment using H_2_O_2_ elimination	[136]
FeSN	A separate investigation devised a novel FeSN, which was produced by doping histidine-doped FeS2 and exhibited significant POD-like activity to eliminate oral biofilm and manage periodontitis	[30]
Au/Pt NCs@GOX	Animal experiments in vivo demonstrated that the Au/Pt NCs@GOX clusterzyme could effectively treat periodontitis in rodents, reduce inflammation, and stimulate the regeneration of periodontal tissue while remaining safe to use	[38]

## Future and landscape of antibacterial nanozymes in dental diseases

Implant restoration is one of the most widely used techniques for replacing missing teeth. The primary benefits are robust retention and the absence of necessity for frequent removals. Peri-implantitis prevention and achieving adequate initial stability are two critical factors that determine the success of implant restorations. This objective can only be attained by commencing with the subsequent two factors. The first step is selecting an appropriate implant. Distinct implant designs exhibit substantial variations in osteogenesis and mechanical strength. For instance, one may opt for implants that possess antibacterial properties or promote osteogenesis [137, 138]. Although the evidence is limited, using locally administered antibiotics alone or in conjunction with nonsurgical or surgical interventions for peri-implantitis demonstrated positive results. Combining systemically administered antibiotics with nonsurgical or surgical interventions remained controversial [139].

In many cases, peri-implantitis is the primary cause of implant failure. Implants may also benefit from the antibacterial properties of nanomaterials. An approach that could be considered is the application of antimicrobial medicines or materials to create a coating that can be modified to impart a significant antibacterial effect onto the implant's surface. An illustration of this can be seen in an in vitro test where ZnNPs modified on the implant surface and prepared as a coating demonstrated a substantial decrease in the quantity of parthenogenic anaerobic bacteria and streptococci in the medium within 96 h, as compared to implants lacking the modified coating. Furthermore, ZnO nanorods and ZnO nanorods were synthesized via the hydrothermal method by Wang et al. After Ti surface modification was applied to ZnO nanorods, ZnNPs and ZnO nanorods were subsequently modified as the outermost layer. The coating can discharge ZnO nanorods expeditiously, and this sustained discharge can impart a dual antibacterial impact. Furthermore, it was demonstrated that the application of CeO NP coating decreased the mean gene expression levels of IL-6, TNF-α, and IL-1b in per Ti tissues, thereby producing a potent anti-inflammatory impact [140–143].

Natural enzymes continue to be superior to NZs, even though the former has been utilized medicinally and as a toothpaste additive for centuries. They are physiologically safe and possess an effective catalytic mechanism, exhibiting high catalytic activity while demineralizing teeth and diminishing dental plaque and calculus [45]. Natural enzymes continue to be superior to NZs, even though the former has been utilized medicinally and as a toothpaste additive for centuries. These substances possess a highly effective catalytic mechanism, exhibit significant catalytic activity, and do not cause any harm to the body while whitening teeth and removing dental plaque and calculus. In contrast, the catalytic mechanism of NZs remains a subject of debate. It defies us how NZs can catalyze enzymes without catalytic activity centers. Consistently, recent research has demonstrated that the fundamental properties of nanomaterials—including size, composition, and shape—and the reaction environment—including temperature, pH, and reactants—are correlated with the catalytic activity of NZs. Changing these characteristics may only provide a partial solution to the issue of insufficient catalytic activity. For instance, metal sulfides are employed as proton-trapping agents to generate H_2_S and expose Fe^3+^, thereby enhancing the efficiency of catalysis [45, 144]. NZs, which possess enzyme-like activity and are multifunctional nanomaterials, have established a presence within the biomedical domain. NZs have made significant contributions to phaseontics and implantology research concerning the maintenance of periodontal health and the enhancement of implant success rates [145].

Since the antimicrobial activity of NZs mainly reliant on their catalytic efficiencies, strategies such as enzyme active center mimicking, downsizing, defect engineering, catalytic processes boosted by external stimulations, and bacterial capture improvement may effectively improve the antimicrobial activity of NZs. In addition to making use of the properties of nanomaterials, NZs may serve as a special foundation for the construction of multifunctional nanoplatforms that combine several antibacterial effects, therefore addressing the limitations of NZ-based CDT on its own in real-world antibacterial treatments. New avenues for creating effective NZs with improved antibacterial activity have been opened up by sophisticated chemical design techniques. Due to the relatively small number of studies conducted so far, these well-chemically engineered NZs still have a long way to go before they are clinically translated. However, they have shown tremendous efficacy in treating bacteria-related illnesses in several in vitro or in vivo models. First off, additional enzymes that may collaborate and take part in the life cycle of microbial diseases are not as well explored as POD, OXD, or hydrolase mimics, which make up the majority of the antibacterial NZs that are now on the market. Investigating novel antibacterial NZs and deciphering their antibacterial processes is crucial. Second, although there have been many efforts to modulate substrate and product selectivity, there have been few attempts to control the catalytic activity of NZs to produce highly effective antibacterial NZs, such as single-atom NZs. Natural enzymes have a large density of active centers, but their distinct three-dimensional spatial architectures are even more crucial in explaining their exceptional specificity and activity. To get highly active and specialized NZs, the purposeful design of enzyme-comparable NZs still needs the cooperative interaction of imitating enzymatic active centers and their spatial microenvironment building. Furthermore, using theoretical stimulations and cutting-edge SAC technologies, primary enzyme-like active sites may be made more distinct and controlled, and the associated catalytic processes can be thoroughly understood in vitro. However, because of the complexity of biological microenvironments, it is difficult to investigate and comprehend the in vivo antibacterial mechanism of NZs. Lastly, even if NZs have shown a promising future in antibacterial therapy, there are still obstacles to overcome before effective disease treatment can be achieved due to their biological safety, possible toxicity, in vivo translocation, biodistribution, degradation, and metabolic pathways, among other issues [146].

Numerous research conducted recently has shown that external stimuli, such as light and ultrasonic waves, may function as a trigger to regulate the activation of NZs. These modes might provide workable solutions to get the intended noticeable site-specificity. One should take biodegradability and biocompatibility into account. Reaching a clinical use beyond the in vivo toxicity of NZs during treatments remains an obstacle. Currently, injecting NZs systemically will unavoidably harm healthy tissues. When it comes to metal-based NZs, the metallic species used to manufacture the NZs is primarily responsible for their toxicity. Although a large body of research has shown the cytoprotective function and biocompatibility of NZs, metal overload-induced metal ion release is still thought to be a potential source of adverse effects on normal tissues. For instance, an excess of copper or iron in healthy tissue or cells may cause a Fenton-like reaction, which might seriously harm nucleic acids and biomacromolecules. As a result, while evaluating the NZs for biocompatibility and biosafety, their pharmacokinetics are crucial. The ability to modify the surface of NZs offers a chance to create biosafety agents. Considering this, surface modification is one of the several approaches to get over the restriction of NZs. Furthermore, considering the ligands of NZs may affect systemic toxicity, clearance kinetics, bioavailability, and therapeutic results. According to this viewpoint, it's crucial to choose a suitable ligand and provide NZs with more biosafety [9, 147].

Although the preponderance of NZ-based therapy systems has broad potential, they lack targeting capabilities [148]. Absorption by healthy cells may result in the induction of toxic side effects in adjacent healthy tissues to different extents. To improve the efficacy of therapy, it may be necessary to administer nanoagents in high doses, which may result in increased tissue toxicity. Due to the factors mentioned above, the development of an innovative NZ system for precise and effective disease treatment is an imperative matter [9, 149]. The interaction between ligands and receptors in living systems can be utilized to develop targeted reagents. After that, pertinent research has progressively surfaced [94]. NZs, which catalyze the conversion of enzyme substrates and exhibit enzymatic kinetics under physiological conditions, are nanomaterials endowed with enzyme-like properties [150].

NZs, being a novel class of synthetic enzymes, offer substitute methodologies for those who rely on enzymatic catalysis. NZs are advantageous over natural enzymes because they are inexpensive, simple to prepare, and stable. These characteristics make NZs prospective for use in various disciplines, including the treatment of antimicrobial infections. Numerous studies have documented the efficacy of NZs in eradicating various resistant pathogenic bacteria, fungi, and viruses and have demonstrated remarkable curative properties against diseases induced by such pathogens [151]. The use of NZs in nanocatalytic medicine is on the rise; these enzymes interact with multifunctional nanomaterials. NZs are considered to be highly effective antibacterial agents due to their extended spectrum of activity and exceptional biocompatibility [152]. The evident potential of NZs to address the drawbacks associated with natural enzymes, including challenges in preparation, denaturation, recycling, and high cost, has been demonstrated. Through biocatalytic processes, NZs have been converted into antibacterial materials of utility. There is a shortage of a comprehensive literature review concerning the use of NZs in the treatment of oral diseases, including but not limited to dental caries, dental pulp diseases, oral ulcers, peri-implantitis, oral cancer monitoring, oral bacteria and ions, soft and hard tissue regeneration [153].

One of the obstacles encountered in the clinical application of nanocatalysis technology for topical oral use has been the requirement to administer two components simultaneously, either through containers with distinct compartments containing each component individually or in two stages (catalytic NP followed by H_2_O_2_ administration). Based on researchers' preliminary testing (unpublished), the latter option, which would permit the mixing of the two constituents before the treatment, is feasible but necessitates custom packaging. Therefore, a one-step nanocatalytic formulation activated under pathological conditions via intrinsic H_2_O_2_ generation could potentially serve as a more viable and precise approach to preventing oral diseases. Additional in vivo investigations into the mechanisms of action and potential toxicity of the bi-functional hybrid NZ would establish the groundwork for clinical implementations aimed at preventing dental caries in humans once its therapeutic precision and efficacy have been established. In the post-microbiome era, when treating polymicrobial diseases necessitates the precise targeting of opportunistic pathogens in mixed communities while preserving the commensals and ecological diversity of the host microbiota, this strategy may be applicable [90, 95].

Natural enzyme mimetics may soon be capable of independently regulating their activities via conformational changes [154]. Diverse methods for modifying the activities of NZs in vitro or in vivo will emerge, thereby broadening their range of applications. NZs present a potentially advantageous resolution to enzyme-related ailments due to their activities approaching those of natural enzymes. Moreover, they may offer distinctive benefits not inherent in natural enzymes [155].

NZs are extensively utilized in clinical disease detection and therapy due to their inherent superiority over natural enzymes. Fer destroys bacteria in situ by binding to the ultrastructure of biofilms, degrading the extracellular polymeric material matrix, and generating free radicals from H_2_O_2_. When combined with modest concentrations of H_2_O_2_, Fer prevents acid injury to mineralized tissue and impedes biofilm formation on authentic teeth in an ex vivo model of biofilm derived from human tissue. The application of Fer and H_2_O_2_ via topical oral therapy in a mouse model of the disease effectively suppresses the progression of dental caries in vivo, thereby mitigating the occurrence of severe tooth decay (cavities). Histological and microbiome analyses indicate no adverse effects observed on the diversity of oral microbiota or gingival and mucosal tissues. The results suggest that Fer could have a novel biomedical application as a topical treatment for prevalent and costly oral diseases caused by biofilms [90]. Researchers used a wearable intraoral device containing FerIONP to treat human participants with implanted natural tooth enamel in a randomized crossover trial. The investigation was carried out under unfavorable circumstances that encourage dental cavities. FerIONP was shown to have a solid antibacterial selectivity against biofilms, including the cariogenic pathogen *S. mutans*, but not against other oral bacteria. Enamel demineralization was significantly reduced as a result. Further studies revealed that FerIONP preferentially eliminated *S. mutans* by producing localized ROS in situ and showed a preferential affinity for the pathogen via a glucan-binding mechanism. Additionally, we proved that FerIONP might be used as a catalyst to identify cariogenic biofilms. Together, Liu et al. provide the first human investigation demonstrating the therapeutic potential of catalytic iron oxide NPs, or NZs, as a targeted nanomedicine for managing an oral infectious illness. To find out whether topical FerIONP with iron or fluoride supplements might boost protective benefits in sensitive people in a synergistic way, further study is needed. Given the established oral-systemic relationship, clinical trials could explore the potential benefits of combining repeated topical oral applications of FerIONP with its systemic use to prevent severe childhood caries and mitigate iron deficiency, which are major unresolved global health issues [156].

Fer, an iron oxide nanoparticle recently approved by the FDA, has been demonstrated to degrade and destroy biofilms that cause dental caries via hydrogen peroxide catalytic activation. Fer, on the other hand, does not affect enamel acid demineralization. The combination of Fe and SnF_2_ inhibits biofilm accumulation and enamel degradation significantly more effectively than either element used alone, according to research. Unexpectedly, the stability of SnF_2_ is improved when it is combined with Fer in aqueous solutions, while the catalytic activity of Fer increases unassisted without the use of any additives. It is worth mentioning that the combination of SnF_2_ and Fer exhibits remarkable efficacy in vivo against dental caries, even at concentrations four times lower, while causing no detrimental effects on the oral microbiome or host tissues. Additionally, comprehensive toxicity investigations are necessary to ascertain the potential long-term consequences of daily application of Fer and SnF_2_. In the context of clinical translation and product development, it may be necessary to optimize the concentrations of Fer, SnF_2_, and H_2_O_2_. However, our findings indicate that Fer and SnF_2_ enhance the therapeutic activity via unforeseen synergistic mechanisms that concurrently target the physicochemical (enamel demineralization) and biological (biofilm) characteristics of dental caries. Moreover, given the frequent association between severe childhood tooth caries and iron deficiency anemia, administering Fer may offer a twofold advantage for these individuals. The potential for utilizing SnF_2_ and Fer to treat anemia and tooth decay, two of the most significant global health issues, presents a viable opportunity to incorporate combination therapy into clinical trials to prevent dental caries in high-risk patients with iron-deficiency anemia. The findings of the researchers demonstrate a powerful therapeutic synergy between approved agents and SnF_2_ stabilization, which can be used to reduce fluoride exposure and prevent a common oral disease [78].Low catalytic activity and an unknown catalytic mechanism Although natural enzymes have been incorporated into toothpaste for therapeutic purposes, they remain more effective than NZs. They can whiten teeth as well as reduce dental calculus and plaque due to their potent catalytic activity, transparent catalytic mechanism, and superior biological safety [157]. However, the catalytic mechanism of NZs remains a subject of debate. Scientists are baffled as to how NZs devoid of catalytic activity centers can imitate the catalytic activity of enzymes. Current research has progressively established a correlation between the catalytic activity of NZs and the fundamental properties of nanomaterials (size, composition, and shape) as well as the reaction environment (pH, temperature, and reactants). Nevertheless, modifying these parameters can only partially mitigate the problem of diminished catalytic activity [158]. Researchers have been working to find effective catalysts and inhibitors for NZs, and they have made some headway in this area. The oxidase, peroxidase, catalase, and superoxide dismutase enzymes that were first used in dental research to demonstrate the enzyme-like properties of NZs were restricted in diversity and lacked substrate specificity. During the alteration process, the researchers added DNase activity. Conversely, NZs are unable to bind to the substrate selectively because they do not have the complex structure of a genuine enzyme–substrate binding bag. They are similar to traditional catalysts in this way [159].

NZs that have been engineered to bind to specific substrates via DNA engineering have demonstrated remarkable efficacy in oral monitoring; however, this does not suffice. Opportunities and obstacles abound in the investigation and implementation of NZs within the field of dentistry. In pursuit of clinical application, scientists must diligently investigate the precise active mechanism of NZs and cultivate additional varieties of NZs to address treatment requirements. Dentists are obligated to propose critically resolvable clinical issues, investigate the molecular biology of the NZ mechanism collectively, and assess potential challenges in the practical implementation of NZs [32].

Although conventional dentistry has been significantly enhanced by revolutionary nanotechnology, there are still several inevitable voids that hinder its complete clinical exploration. In comparison to other areas of biological research, the current state of nanodentistry research is belated. The advancement of cost-effective and efficient nanotheranostics will be facilitated by the increased focus on patient needs in research [160]. The pharmacokinetics, absorption, metabolism, biodistribution, therapeutic duration, excretion, and toxicity of NZs need to be explored and understood at different phases of administration and therapy. According to several data on biodistribution, non-targeting NZs tend to accumulate in the lung, liver, and spleen. The primary factors influencing the progress of NZ clinical use will be their therapeutic safety and biocompatibility [56].

Although scientists have created a variety of surface-modified NZs using polymers, nucleic acids, and antibodies to imitate natural enzyme selectivity, the resulting selectivity is still inadequate for usage in real-world applications. Regarding environmental and medicinal uses, the toxicity of NZs to people and the environment is another crucial problem that has to be resolved. Future research will employ a strategy of rational screening of enzyme-like activity based on those atomic compositions which are envisaged to catalyze enzymatic reactions, in contrast to traditional research on developing NZs, which has been carried out by random screening of the enzyme-like activities of existing unspecified nanomaterials. Furthermore, by using their synergistic impact to promote electron transport across composite materials during redox reactions, a technique to produce composites should be able to overcome the primary limits that now exist with NZs of poor catalytic activity. By successfully avoiding the use of hazardous chemicals in traditional chemical synthesis, bioinspired synthesis of NZs also offers a way to manufacture benign NZs, speeding up their usage in therapeutic applications. Lastly, the sector will benefit from the development of innovative surface engineering techniques that can make NZs specific to target substrates [161]. Given the aforementioned research initiatives, we anticipate that nanoenzymes will soon be extensively used in a variety of dental infection treatments.

## Conclusions

The escalating global incidence of periodontal and peri-implant diseases has garnered considerable interest. NZs, which possess enzyme-like activity and are multifunctional nanomaterials, have established a presence within the biomedical domain. NZs have significantly advanced research in the fields of periodontics and implantology, specifically about the maintenance of periodontal health and the enhancement of implant success rates. Natural enzymes have inherent drawbacks that are circumvented by NZs, including but not limited to poor environmental stability, high production costs, and storage difficulties. The development of dentistry is parallel to that of material science. Oral NZ research and utilization is emerging as a distinct subfield within nanocatalytic medicine. To underscore the significant impact that NZs have on dental health, we began by conducting a comprehensive review of the overall research advancements concerning multifunctional NZs about oral diseases. This included the treatment of dental caries, pulp diseases, oral ulcers, and peri-implantitis; the monitoring of oral cancer, oral bacteria, and ions; and the regeneration of both soft and hard tissue. In addition, the investigation and application of NZs in dentistry face a multitude of potential obstacles. Scientists must make a concerted effort to fully comprehend the precise energetic mechanism of NZs and develop novel varieties of NZs to satisfy the therapeutic demand for experimental clinical application. Urgent clinical concerns must be attended to, dental researchers must collaborate to comprehend NZ mechanisms at the molecular biology level, and they must assess potential complications in NZ application. As in vitro and in vivo research increased, we encountered a dearth of data-driven significance in the extraction data. It is highly recommended that future experimental investigations utilize a more substantial sample size, and that additional systematic assessments be undertaken to evaluate the effectiveness of these scientific experiments. Consequently, it is crucial to address the unresolved challenges through concerted efforts; doing so will significantly advance future research endeavors. It is expected that this extensive examination will not only increase the interest and enthusiasm of researchers in the domain of NZs but also furnish them with invaluable knowledge and perspectives that aid in the investigation of catalytic mechanisms intrinsic to NZs that have yet to be explored. Hence, the aforementioned obstacles symbolize the vanguard of forthcoming NZ research, compelling additional inquiry and scrutiny.

## Data Availability

Not applicable.
