# Editorial Expression of Concern: The potential use of nanozymes as an antibacterial agents in oral infection, periodontitis, and peri-implantitis

**DOI:** 10.1186/s12951-025-03391-1

**Published:** 2025-05-02

**Authors:** Mohammad Hosseini Hooshiar, Ashkan Badkoobeh, Shirin Kolahdouz, Azadeh Tadayonfard, Asieh Mozafari, Kamyar Nasiri, Sara Salari, Reza Safaralizadeh, Saman Yasamineh

**Affiliations:** 1 Department of Periodontology, School of Dentistry, Tehran University of Medical Sciences, Tehran, Iran; 2 Department of Oral and Maxillofacial Surgery, School of Dentistry, Qom University of Medical Sciences, Qom, Iran; 3 School of Dentistry, Shahid Sadoughi University of Medical Sciences, Yazd, Iran; 4 Postgraduate Department of Prosthodontics, Dental Faculty, Tehran University of Medical Sciences, Tehran, Iran; 5 Department of Periodontics, Faculty of Dentistry, Qazvin University of Medical Sciences, Qazvin, Iran; 6 Department of Dentistry, Islamic Azad University of Medical Sciences, Tehran, Iran; 7 Islamic Azad University of Medical Sciences, Esfahan, Iran; 8 Restarative Dentistry, Department of Dental, Faculty Tabriz Medical University, Tabriz, Iran; 9 Young Researchers and Elite Club, Tabriz Branch, Islamic Azad University, Tabriz, Iran

The Editor-in-Chief is issuing an Editorial Expression of Concern to alert readers that this article underwent unusual changes to the authorship list during the submission process. Readers are advised to interpret the contributions of the authors to this article with caution. Saman Yasamineh has stated on behalf of the authors that they disagree to this Expression of Concern.

